# Common genetic variants do not impact clinical prediction of methotrexate treatment outcomes in early rheumatoid arthritis

**DOI:** 10.1111/joim.20087

**Published:** 2025-04-06

**Authors:** Anton Öberg Sysojev, Bénédicte Delcoigne, Thomas Frisell, Lars Alfredsson, Lars Klareskog, The SRQ biobank group, Saedis Saevarsdottir, Magnus Boman, Leonid Padyukov, Johan Askling, Helga Westerlind

**Affiliations:** ^1^ Clinical Epidemiology Division Department of Medicine Solna Karolinska Institutet Stockholm Sweden; ^2^ Institute of Environmental Medicine (IMM) Karolinska Institutet Stockholm Sweden; ^3^ Division of Rheumatology Department of Medicine Solna Karolinska Institutet and Karolinska University Hospital Stockholm Sweden; ^4^ Center for Molecular Medicine Department of Medicine Solna Karolinska Institutet Stockholm Sweden; ^5^ Faculty of Medicine School of Health Sciences University of Iceland Reykjavik Iceland; ^6^ MedTechLabs BioClinicum Karolinska University Hospital Stockholm Sweden; ^7^ Rheumatology, Theme Infection and Inflammation Karolinska University Hospital Stockholm Sweden

**Keywords:** machine learning, methotrexate, pharmacogenetics, polymorphism, rheumatoid arthritis, single nucleotide

## Abstract

**Background:**

Methotrexate (MTX) is the mainstay initial treatment of rheumatoid arthritis (RA), but individual response varies and remains difficult to predict. The role of genetics remains unclear, but studies suggest its importance.

**Methods:**

Incident RA patients starting MTX‐monotherapy were identified through a large‐scale Swedish register linkage. Demographic, clinical, medical, and drug history features were combined with fully imputed genotype data and used to train and evaluate multiple learning models to predict key MTX treatment outcomes.

**Results:**

Among 2432 patients, we consistently observed an estimated area under the curve (AUC) of ∼0.62, outperforming models trained on sex and age. The best performance was observed for EULAR primary response (AUC = 0.67), whereas models struggled the most with predicting discontinuation. Genetics provided negligible improvements to prediction quality.

**Conclusions:**

Despite an extensive study population with broad multi‐modal data, predicting MTX treatment outcomes remains a challenge. Common genetic variants added minimal predictive power over clinical features.

## Introduction

Although current treatment guidelines recommend starting patients newly diagnosed with rheumatoid arthritis (RA) on methotrexate (MTX) [1], actual treatment response and subsequent outcome remain highly variable [2]. To facilitate early identification of treatment responders, recent attention has been given to the development of machine learning models supporting the prediction of MTX treatment outcomes among patients with RA. Although results have so far been promising, studies have primarily been conducted in limited samples of only a few hundred patients, the majority of which lack sufficient external validation of their findings (Table S1) [3, 4, 5, 6, 7, 8, 9, 10, 11]. Furthermore, these models have primarily been trained solely on clinical features, despite promising performance from a handful of prediction models trained additionally on genetic data [9, 10] and indications of a modest heritability of MTX treatment response [12, 13]. As such, more evidence is needed from studies in larger cohorts, including both traditional clinical variables and genetic information, using robust and replicable pre‐processing of data before widespread implementation within routine rheumatological practice can be considered. To this end, we here leveraged large‐scale data from a Swedish cohort of early RA patients to train and evaluate machine learning models in predicting key MTX treatment outcomes. Within this setting, we thoroughly explored the impact of common single‐nucleotide polymorphisms (SNPs) on prediction quality—over and above that of baseline clinical data—and used the underlying data sources to evaluate results from previous studies.

## Methods and materials

### Data materials

Our cohort consisted of Swedish early RA patients starting MTX in monotherapy (i.e., not combined with another disease‐modifying anti‐rheumatic drug, DMARD), with available genotype data through the Epidemiological Investigation of RA (EIRA) study or the Swedish Rheumatology Quality Register (SRQ) biobank (SRQb) (Supporting Information section). Early RA was defined as having no more than 12 months of symptom duration at the time of RA diagnosis. First‐line treatment with MTX in DMARD‐monotherapy was ascertained by including only patients starting MTX as their first DMARD, without additional non‐MTX DMARDs prescribed within 30 days following their first MTX prescription. Treatment outcomes included persistence to treatment with MTX at one and 3 years after treatment start, respectively (i.e., remaining adherent by the end‐point without initiating additional non‐MTX DMARDs during follow‐up), along with primary response at 6 months (i.e., EULAR good or moderate contra no response), DAS28‐remission at 12 months, and discontinuation of MTX, as well as reaching DAS28‐remission by 6 months and remaining on MTX by 1 and 3 years, respectively, with a particular focus on the outcome of persistence. All decisions and measures related to the target outcomes were made by the treating rheumatologist.

For training, we employed a broad set of features covering different aspects of patient information spanning the 5 years preceding treatment initiation (Supporting Information section). Specifically, data on demographics, clinical variables, medical history, and drug prescriptions were extracted from multiple nationwide registers, all of which were derived blinded to the outcome. Genotype data on common genetic variants were retrieved from EIRA and SRQb, genotyped on the Illumina Infinium Global Screening Array at deCODE Genetics Inc., and subsequently imputed to improve coverage.

### Data pre‐processing

**Fig. 1:**
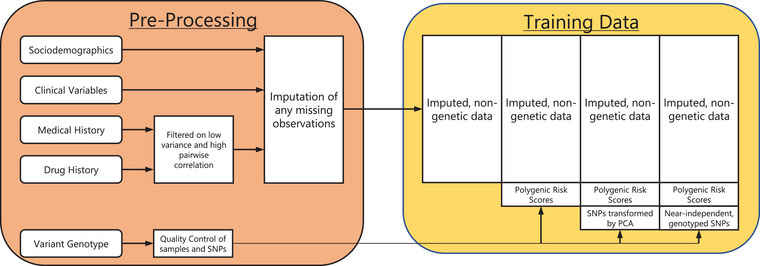
**Data‐processing flowchart**. Flowchart visualization of the different data modalities included into the study, and the different types of training sets which they contribute toward. Left part represents the raw data, as well as its pre‐processing and subsequent aggregation; right part represents the four distinct sets of training data, used throughout this study to train the multiple learning models.

To ascertain the robustness of the training data, we subjected our features to thorough cleaning and quality control (Fig. 1 and Supporting Information section). In short, we first filtered out medical history and drug prescription features with low observed prevalence and high pairwise concordance, subsequently imputing missing observations in demographic and clinical features with the full set of remaining non‐genetic features, using a single imputation through a random forest approach, as implemented in the R package “missforest” [14] (Tables S2–S5). Next, fully imputed genotype data were subjected to a standard genome‐wide association study quality control, filtering on the quality of both samples and SNPs (Table S6). Two approaches to dimensionality reduction were then employed: the first using principal component analysis to transform the remaining SNPs into a latent representation of the full set of variants and the second using linkage disequilibrium pruning among directly genotyped SNPs to derive a subset of near‐independent variants. Additionally, we used the set of variants remaining after quality control to construct polygenic risk scores (PRSs) for multiple traits. In particular, we constructed a PRS based on known genetic risk factors for RA, along with PRSs for traits with a putative relation to MTX treatment response per a previous study from our group [15] and for various established RA comorbidities, risk factors, and treatment response predictors (Table S7).

### Supervised learning

Supervised learning was performed in four overlapping feature sets (Fig. 1). All sets included the non‐genetic data (i.e., demographics, clinical variables, medical history, and drug prescription data), with the second set additionally incorporating PRSs as individual features; the third set also included the derived principal components, whereas the fourth set instead included the set of near‐independent genotyped variants. To avoid optimism and reduce the risk of overfitting, training was done through nested cross‐validation (Fig. S1), tuning hyperparameters within the inner loop (Supporting Information section) and evaluating models within the outer loop. Four distinct machine learning models were employed, including two regression models (logistic and elastic net) and two tree‐based methods (random forest and extreme gradient boosting, XGboost).

Model performance was quantified by the area under the curve (AUC), whereas classification differences across the four sets of training data were assessed through total and net reclassification improvement [16]. The best performing model was re‐fit using the full training data and subsequently taken forward for feature importance analysis as quantified through Shapley Additive Explanations (SHAP), using a kernel‐based approach as implemented in the R package “kernelshap” [17]. Additional feature importance was assessed by contrasting distributional differences in non‐genetic characteristics across individuals correctly classified with high probability, that is, probability in the top decile for each label (Supporting Information section). Sensitivity toward the high‐probability grouping was assessed by repeating the analysis using a more lenient grouping of the top three deciles.

### Secondary analyses

We performed multiple secondary analyses to further explore model performance across features, outcomes, and populations. First, we evaluated two additional models, one trained solely on sex and age and one trained on sex, age, and the previously derived set of near‐independent genotyped SNPs. Second, we retrained models within sub‐cohorts of seropositive and seronegative RA patients, classifying patients positive for rheumatoid factor or anti‐citrullinated peptide antibodies as seropositive and those negative for both as seronegative. Third, we evaluated two published prediction models within our own study cohort [9, 10] by creating feature sets replicating those employed previously and training models to predict both the treatment outcomes of the corresponding studies and our persistence outcomes (Supporting Information section).

### Ethical approval

This study was approved by the Stockholm ethical review board (DNR 96–174, DNR 2006/476‐31/4, DNR 2012/2070‐31/2). Patients were not involved in any stage of the study, and informed consent was thus not obtained, as establishing contact with patients would have limited the available study population to a biased selection while also infringing on patient privacy. Nevertheless, all analyses were performed on pseudonymized data, securely stored at encrypted local servers, with access limited solely to involved researchers.

## Results

Our cohort consisted of 2387 early RA patients treated with MTX as their first‐ever DMARD, of which 1635 (67%) were persistent at 1 year and 1029 (45%) were persistent at 3 years, with data on 105 non‐genetic features, 17 PRSs, 6 principal component representations of the full set of genotypes, and ∼16,000 genotyped near‐independent variants. In training on non‐genetic features, performance was stable across all algorithms except logistic regression, achieving AUCs of ∼0.62 for both persistence outcomes (Table 1), thereby outperforming models trained on sex and age alone for both persistence at 1 (AUC = 0.587, 95%CI 0.562–0.611) and 3 years (AUC = 0.613, 95%CI 0.590–0.636). The inclusion of genetic data resulted in only minimal changes to predictive quality (Table 1), also supported by the reclassification statistics (Table S8) and by AUCs from models trained on sex, age, and the set of near‐independent genotyped variants (AUC = 0.589, 95%CI 0.565–0.614 and AUC = 0.616, 95%CI 0.593–0.639, for persistence at 1 and 3 years, respectively). Predictive quality was generally similar in subsets of seropositive and seronegative patients (Table S9), as well as in most of the additional treatment outcomes, where the best performance was observed in predicting primary treatment response at 6 months (AUC = 0.666, 95%CI 0.604–0.728), whereas models struggled the most in predicting discontinuation (Table S10).

**Table 1:** Model prediction quality.

	Non‐genetic characteristics	Non‐genetic characteristics, and PRS	Non‐genetic characteristics, PRS, and principal components	Non‐genetic characteristics, PRS, and near‐independent genotyped variants
**Persistence at 1 year, *N* = 1635 (67%)**				
*Logistic regression, AUC (95%CI)*	0.598 (0.544–0.652)	0.598 (0.544–0.653)	0.594 (0.539–0.648)	–
*Elastic net regression, AUC (95%CI)*	0.618 (0.564–0.675)	0.621 (0.568–0.675)	0.616 (0.562–0.670)	0.602 (0.547–0.656)
*Random forest, AUC (95%CI)*	0.612 (0.559–0.666)	0.621 (0.567–0.675)	0.617 (0.563–0.671)	0.625 (0.571–0.679)
*XGBoost, AUC (95%CI)*	0.620 (0.567–0.674)	0.619 (0.566–0.673)	0.618 (0.564–0.672)	0.539 (0.483–0.594)
**Persistence at 3 years, *N* = 1029 (45%)**				
*Logistic regression, AUC (95%CI)*	0.602 (0.551–0.654)	0.600 (0.549–0.652)	0.595 (0.543–0.647)	‐
*Elastic net regression, AUC (95%CI)*	0.625 (0.574–0.676)	0.627 (0.576–0.677)	0.624 (0.574–0.675)	0.623 (0.572–0.674)
*Random forest, AUC (95%CI)*	0.621 (0.569–0.672)	0.612 (0.561–0.663)	0.615 (0.563–0.666)	0.615 (0.564–0.666)
*XGBoost, AUC (95%CI)*	0.607 (0.555–0.658)	0.606 (0.555–0.658)	0.605 (0.554–0.657)	0.563 (0.510–0.615)

*Note*: Model quality in predicting persistence to treatment with MTX at 1 and 3 years after treatment start, respectively, for four distinct models, trained on four distinct sets of training features. Estimates of AUC are averaged across the cross‐validation folds; confidence intervals are computed through 2000 stratified bootstrap replicates.

**Fig. 2:**
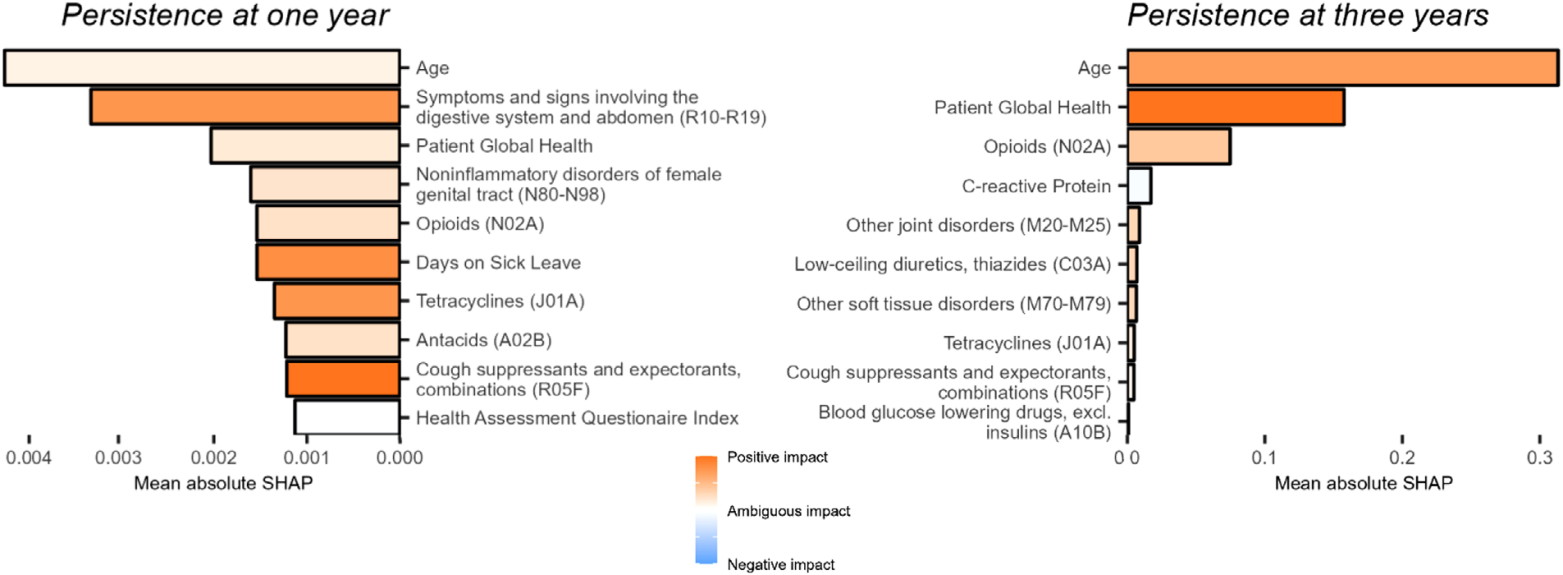
**Global feature importance through Shapley Additive Explanations (SHAP)**. Top 10 features with the highest mean absolute SHAP, for the models trained on non‐genetic characteristics and polygenic risk scores (PRSs), using elastic net regression.

Using the feature set consisting of non‐genetic characteristics and PRSs, we retrained elastic net regression models with the complete data for subsequent analysis of feature importance with respect to the two persistence outcomes. Numerical assessment of calibration quality indicated no issue for either of the two outcomes (Table S11), though visual inspection of the calibration plots suggested a poor spread of probabilities. As such, we recalibrated probabilities prior to feature importance analysis, finding that beta calibration resulted in the most stable probabilities (Figs. S2 and S3). Global SHAP feature importance identified age and patient global health as two of the most important predictors for both outcomes (Fig. 2). This was observed also when contrasting patients correctly labeled with high probability, which further indicated that non‐persistent patients reported higher pain, scored higher in HAQ, and were more often women (Table S12). Results were similar when a more lenient threshold for considering patients correctly classified was employed (Table S13).

In validating the model of Lim et al. [9], we observed an AUC = 0.522 (95%CI 0.467–0.578) in predicting attainment of DAS28 < 2.6 within 2 years of treatment start, with a slightly higher AUC observed in predicting both persistence at 1 (AUC = 0.549, 95%CI 0.494–0.604) and 3 years (AUC = 0.587, 95%CI 0.535–0.639). Similarly, upon validating the model of Myasoedova et al. [10], we obtained an AUC = 0.625 (95%CI 0.566–0.684) in predicting primary response at 3 months, with similar but somewhat attenuated performance in predicting persistence (AUC = 0.588, 95%CI 0.533–0.643 and AUC = 0.609, 95%CI 0.557–0.660, for persistence at 1 and 3 years, respectively).

## Discussion

In this study, we used the so far most comprehensive set of multi‐modal data on demographic, clinical, medical history, and drug prescription features, in combination with genome‐wide genotype data on common genetic variants, to predict key clinical treatment outcomes. Despite an extensive cohort and a broad feature set, overall predictive capacity was weak, with AUC point estimates consistently around 0.60–0.67, estimates that were only minimally impacted by the addition of genetic data. Although previous studies have reported AUCs of ∼0.75 using features corresponding to a subset of those here referred to as demographics and clinical variables, within training samples less than a fourth the size of the current sample, we were here unable to achieve comparable performance [4, 5, 7]. Nevertheless, in validating two previous studies exhibiting high predictive performance, we obtained AUCs similar to or worse than those observed within our primary analyses, suggesting that the optimistic AUCs of previous studies may be due to overfitting, a likely result of model development in samples too small for the set of features considered [18].

Among individual features, age at diagnosis and patient global health at baseline were among the strongest predictors of both persistence outcomes, corroborating findings from previous studies [4, 7, 10]. Having recently been prescribed opioids, tetracyclines, or cough suppressants and expectorants was also recognized as important across both outcomes, treatments that may be indicative of a greater general frailty or serve as proxies for low tolerance to typical treatment side effects such as gastrointestinal problems or headaches. Notably, we did not identify baseline DAS28 as an influential predictor, in contrast to previous studies [4, 5, 7, 10], though it is worth noting that these studies relied on short‐term follow‐up for outcomes defined by various dichotomizations of DAS28, wherein baseline measures would inevitably be strong predictors of the outcome. Notably, neither of the genetic features, including the RA PRS, were observed among the most important features for either persistence outcome.

The major strength of our study lies in the data employed, exceeding most previous studies in sample size, follow‐up duration, cohort homogeneity, and breadth of available information. Nevertheless, our study also had limitations. First, despite wide coverage of training features, we lacked data on BMI, one of the few variables consistently shown to be associated with MTX treatment response [19]. Although measured BMI is available for participants of EIRA, its systematic missingness from SRQb made us wary about its inclusion here, despite thorough imputation of missing observations. Second, although our focus was specifically on common variants from SNP array genotyping and subsequent imputation, other genetic factors not considered here may yet prove relevant in predicting MTX treatment response [20].

Although our study found all trained models to robustly outperform chance, performance was generally weaker than in preceding studies from smaller cohorts, indicating that these have likely suffered from overfitting. Feature importance analyses suggested several clinical and demographic features as most influential, identifying patients diagnosed at a young age and patients reporting poor baseline global health as being particularly prone to non‐persistence. Genetic data, as modeled here, provided only a minimal contribution to overall model performance when added on top of non‐genetic data. Although the clinical utility of successful prediction of MTX treatment outcome remains apparent, our results demonstrate that more work in large cohorts is needed before machine learning will meaningfully add decision‐making support over and above what is currently available for the rheumatologist.

## Author contributions

All authors contributed to the conception and design of the study. Bénédicte Delcoigne, Thomas Frisell, Lars Klareskog, Lars Alfredsson, Saedis Saevarsdottir, Leonid Padyukov, Johan Askling, Helga Westerlind, Anton Öberg Sysojev, and “the SRQ Biobank Group” generated raw data by collecting study participants (Lars Klareskog, Lars Alfredsson, Saedis Saevarsdottir, Leonid Padyukov, Johan Askling, Helga Westerlind, and “the SRQ Biobank Group”), coordinating genotyping of study participants (Saedis Saevarsdottir), imputation of non‐genotyped variants (Anton Öberg Sysojev), and register linkage (Bénédicte Delcoigne, Thomas Frisell, Johan Askling). Anton Öberg Sysojev performed the statistical analysis along with the drafting of the manuscript. All authors interpreted the data and revised the manuscript for important intellectual content. All authors approved the final version of the manuscript for publication.

## Conflict of interest statement

Karolinska Institutet has entered into agreements with the following companies with J.A. as PI: Abbvie, BMS, Eli Lilly, Galapagos, Janssen, Pfizer, Roche, Samsung Bioepis, and Sanofi. S.S. is an employee at deCODE genetics Inc. The authors declare no other conflicts of interest.

## Funding information

J.A. was supported by the Swedish Research Council, Nordforsk, Vinnova, Region Stockholm/Karolinska Institutet Funds (ALF), the Swedish Cancer Society, and the Swedish Heart Lung Foundation. H.W. received support for the project from Reumatikerförbundet, Stiftelsen Professor Nanna Svartz Fond, Stiftelsen Konung Gustaf V:s 80‐årsfond, Karolinska Institutet foundations. M.B. was supported by the Swedish Research Council. T.F. was supported by the Swedish Research Council (2021‐01418). This project was supported by Vinnova, Innovationsfonden, and the Research Council of Norway under the frame of Nordforsk (Grant No. 90825, Project NORA) and by the Swedish Research Council, the Federal Ministry of Education and Research in Germany, and the Research Council of Norway, under the frame of ERA PerMed (Project ScandRA).

## Supporting information




**Table S1: Previously published literature on prediction of MTX treatment response**. *Results from a literature search conducted in the Spring of 2024, targeting English‐language papers indexed through PubMed, on rheumatoid arthritis AND machine learning AND methotrexate, all as MeSH terms*.
**Table S2: Demographic features, within the study cohort**. *As measured at baseline, prior to imputation of missing data and standardization of features, with categorical variables represented by counts, and quantitative variables as medians. Missing observations were excluded from computations of these statistics*.
**Table S3: Clinical features, within the study cohort**. *As measured at baseline, prior to imputation of missing data and standardization of features, with categorical variables represented by counts, and quantitative variables as medians. Missing observations were excluded from computations of these statistics*.
**Table S4: Medical history features, within the study cohort**. *As described by the occurrence of ICD‐10 codes from the five years preceding the first prescription of MTX, remaining after filtering on low variance and high concordance*.
**Table S5: Drug prescription features, within the study cohort**. *As described by the occurrence of four‐character ATC‐codes from the year immediately preceding the first prescription of MTX, remaining after filtering on low variance and high concordance*.
**Table S6: Quality control procedure**. *Summary of the samples and variants removed during the quality control procedure for the genotype data in the study cohort*.
**Table S7: Phenotypes for which UK Biobank GWAS data were extracted and employed in the creation of PRSs**.
**Table S8: Reclassification statistics**. *Net reclassification and total reclassification for both persistence at one year and persistence at three years, assessed for each of the four prediction algorithms, when adding each set of genetic features to the non‐genetic characteristics*.
**Table S9: Model prediction quality in seropositive and seronegative patients**. *Model quality in predicting persistence to treatment with MTX at one and three years after treatment start, respectively, for four distinct models, trained on four distinct sets of training features, in patients with seropositive and seronegative RA, respectively*. *Estimates of AUC are averaged across the cross‐validation folds; confidence intervals are computed through 2000 stratified bootstrap replicates*.
**Table S10: Model prediction quality towards alternative treatment outcomes**. *Model quality in predicting alternative MTX treatment outcomes for four distinct models, trained on four distinct sets of training features*. *Estimates of AUC are averaged across the cross‐validation folds; confidence intervals are computed through 2000 stratified bootstrap replicates*.
**Table S11: Demographic and clinical features at baseline, for patients correctly classified with high probability**. *Classifications were based on cut‐offs suggested as optimal from maximizing Youden's index, with high probability defined as those in the top/bottom decile of predicted probabilities. P‐values were obtained by t‐tests for numerical variables and χ^2^‐tests for categorical variables*.
**Table S12: Demographics and clinical features at baseline, for those correctly classified with high probability**. *Classifications were based on cut‐offs suggested as optimal in maximizing Youden's index, with high probability defined as those in the top/bottom three deciles of predicted probabilities. P‐values were obtained by t‐tests for numerical variables and χ^2^‐tests for categorical variables*.
**Table S13: Refitted prediction model quality**. *Model quality measures in predicting persistence at one and three years, respectively, based on elastic net regression trained with non‐genetic characteristics and PRS; AUC confidence intervals are computed through 2000 stratified bootstrap estimates*.
**Figure S1: Supervised learning pipeline**. *Flowchart visualization of the nested cross‐validation loop as implemented in the underlying supervised learning pipeline. The green box visualizes the outer loop, whereas the blue box visualizes the inner loop, repeated within each of the five segments observable within the green box*.
**Figure S2: Graphical visualization of model calibration for persistence at one year**. *Flexible loess‐smoothed curve of fitted probabilities on observed labels, with each point and error bar representing deciles of fitted probabilities. Diagonal line represents expected distribution. Both models are trained with elastic net regression*.
**Figure S3: Graphical visualization of model calibration for persistence at three years**. *Flexible loess‐smoothed curve of fitted probabilities on observed labels, with each point and error bar representing deciles of fitted probabilities. Diagonal line represents expected distribution. Both models are trained with elastic net regression*.

## Data Availability

For reasons related to the ethical and legal permits surrounding the individual study data, these cannot be shared publicly; requests pertaining to data access can be directed to the EIRA secretariat (http://www.eirasweden.se) and the SRQ biobank (https://srq.nu/en/). Summary statistics and code are made available via the first author's GitHub page.

## Appendix A

**Swedish Rheumatology Quality Register Biobank Group authors**: Members of the Swedish Rheumatology Quality Register Biobank Group are as follows: **Johan Askling**, Clinical Epidemiology Division, Department of Medicine Solna, Karolinska Institutet, Stockholm, Sweden; **Eva Baecklund**, Department of Medical Sciences, Rheumatology, Uppsala University, Uppsala, Sweden; **Lena Björkman**, Department of Rheumatology and Inflammation Research, University of Göteborg, Göteborg, Sweden; **Alf Kastbom**, Department of Biomedical and Clinical Sciences, Linköping University, Linköping, Sweden; **Solbritt Rantapää‐Dahlqvist**, Department of Public Health and Clinical Medicine, Rheumatology, Umeå University, Umeå, Sweden; **Carl Turesson**, Department of Clinical Sciences, Malmö, Lund University, Malmö, Sweden.
