# Supplementary material for: Common genetic variants do not impact clinical prediction of methotrexate treatment outcomes in early rheumatoid arthritis
Source: J Intern Med. 2025 Apr 6;297(6):693–701. doi: 10.1111/joim.20087 (PMC12087826; doi:10.1111/joim.20087)
Supplement: Supplementary file 1 — Table S1: Previously published literature on prediction of MTX treatment response. Results from a literature search conducted in the Spring of 2024, targeting English‐language papers indexed through PubMed, on rheumatoid arthritis AND machine learning AND methotrexate, all as MeSH terms. Table S2: Demographic features, within the study cohort. As measured at baseline, prior to imputation of missing data and standardization of features, with categorical variables represented by counts, and quantitative variables as medians. Missing observations were excluded from computations of these statistics. Table S3: Clinical features, within the study cohort. As measured at baseline, prior to imputation of missing data and standardization of features, with categorical variables represented by counts, and quantitative variables as medians. Missing observations were excluded from computations of these statistics. Table S4: Medical history features, within the study cohort. As described by the occurrence of ICD‐10 codes from the five years preceding the first prescription of MTX, remaining after filtering on low variance and high concordance. Table S5: Drug prescription features, within the study cohort. As described by the occurrence of four‐character ATC‐codes from the year immediately preceding the first prescription of MTX, remaining after filtering on low variance and high concordance. Table S6: Quality control procedure. Summary of the samples and variants removed during the quality control procedure for the genotype data in the study cohort. Table S7: Phenotypes for which UK Biobank GWAS data were extracted and employed in the creation of PRSs. Table S8: Reclassification statistics. Net reclassification and total reclassification for both persistence at one year and persistence at three years, assessed for each of the four prediction algorithms, when adding each set of genetic features to the non‐genetic characteristics. Table S9: Model prediction quality in seropositive a [file JOIM-297-693-s001.docx]

**SUPPLEMENTARY MATERIAL FOR “COMMON GENETIC VARIANTS DO NOT IMPACT CLINICAL PREDICTION OF METHOTREXATE TREATMENT OUTCOMES IN EARLY RHEUMATOID ARTHRITIS”**

**Anton Öberg Sysojev^1^, PhD; Bénédicte Delcoigne^1^, PhD; Thomas Frisell^1^, PhD; Lars Alfredsson^2^, PhD; Lars Klareskog^3,4^, MD, PhD; the SRQ biobank group, Saedis Saevarsdottir^1,5^, MD, PhD; Magnus Boman^1,6^, PhD; Leonid Padyukov^3,4^, PhD; Johan Askling^1,7^, MD; PhD; Helga Westerlind^1^, PhD.**

^1^Clinical Epidemiology Division, Department of Medicine Solna, Karolinska Institutet, Stockholm, Sweden.

^2^Institute of Environmental Medicine (IMM), Karolinska Institutet, Stockholm, Sweden.

^3^Division of Rheumatology, Department of Medicine Solna, Karolinska Institutet and Karolinska University Hospital, Stockholm, Sweden.

^4^Center for Molecular Medicine, Department of Medicine Solna, Karolinska Institutet, Stockholm, Sweden.

^5^Faculty of Medicine, School of Health Sciences, University of Iceland, Reykjavik, Iceland.

^6^MedTechLabs, BioClinicum, Karolinska University Hospital, Stockholm, Sweden.

^7^Rheumatology, Theme Infection and Inflammation, Karolinska University Hospital, Stockholm, Sweden.

Corresponding author: Anton Öberg Sysojev; Clinical Epidemiology Division, Eugeniahemmet, T2, Karolinska Universitetssjukhuset, Solna 171 76, Stockholm, Sweden; anton.oberg.sysojev@ki.se; +1(0) 347-273-9397; 0000-0003-3221-7979.

**TABLE OF CONTENTS**

**SUPPLEMENTARY NOTE	(3-8)**

**Study cohort**

**Register linkage data**

**Data pre-processing and quality control**

**Supervised learning parameter tuning**

**Alternative feature importance**

**Evaluation of previously published models trained on genetic data**

**SUPPLEMENTARY TABLES	(9-20)**

**SUPPLEMENTARY FIGURES	(21-23)**

**REFERENCES	(24)**

**SUPPLEMENTARY NOTE**

**Study cohort**

The study cohort was extracted from the Epidemiological Investigation of Rheumatoid Arthritis (EIRA) study and the Swedish Rheumatology Quality register (SRQ) biobank (SRQb) study, by including early rheumatoid arthritis (RA) patients receiving methotrexate (MTX) in DMARD-monotherapy as their first RA treatment in 2006 or later, with available genotype data [1]. EIRA was established in 1996 and has since enrolled both newly diagnosed RA patients from the middle and southern regions of Sweden, as well as RA-free matched controls. Thorough and extensive questionnaire data is available for each participant from the point of inclusion, with follow-up questionnaire data available for a subset [2]. The SRQ is a nationwide quality register established during the mid-90s, used by most rheumatologists in Sweden to monitor the disease course of patients as part of standard care. It has an estimated coverage >85% for all prevalent RA patients in Sweden, with extensive baseline and longitudinal information on clinical variables and prescribed drugs [3, 4]. The SRQb was initiated in 2012 as a research project adjunct to SRQ, enrolling patients consenting to donation of blood samples, who are invited upon receiving an incident RA diagnosis or at initiation of a biological or targeted-synthetic DMARD.

**Register linkage data**

Training data primarily comes from a large-scale register linkage, connecting our study cohort of early RA patients initiating MTX in DMARD-monotherapy as first-line treatment, to various Swedish nationwide registers, through the use of their personal identification number [5]. Here, we use data from the Total Population Register, the Longitudinal Integration Database for Health Insurance and Labor Market Studies, the Social Insurance Agency, the Multi-Generation Register, the National Patient Register and the Prescribed Drug Register. More specifically: the Total Population Register primarily contains data on population demographics [6], from which we extract information on date of birth, country of birth and sex; the Longitudinal Integration Database for Health Insurance and Labor Market Studies contains data on aspects such as income, education and occupation [7], from which we identify information on highest attained educational level at baseline; the Social Insurance Agency contains information relating to social insurance, from which we extract data on sick leave and disability pension; and the Multi-Generation Register links individuals to their relatives [8], which we use to identify first-degree relatives with an RA-diagnosis. The National Patient Register contains all inpatient hospitalizations occurring since 1987 in Sweden [9], with additional data on outpatient hospitalizations from 2001 and onwards, from which we extracted all primary ICD-10 codes for inpatient and specialist outpatient visits reported within the five years preceding RA diagnosis, subsequently grouping them on subchapter level (e.g., M05-M14 for inflammatory polyarthritis, D10-D36 for benign neoplasms). The Prescribed Drug Register contains all expediated drug prescriptions made since 2005 and onwards, from which we extracted all ATC-codes reported within the year immediately preceding RA diagnosis, subsequently grouping them by the first four characters of the corresponding code [10].

Raw genotype data was available for a subset of participants. Biological samples from consenting participants of EIRA and SRQb were subsequently taken forward for genotyping, conducted at deCODE Genetics Inc., Reykjavik, Iceland, on the Illumina Infinium Global Screening Array. Raw genotype data was subsequently imputed to obtain additional information on variants not-directly assayed after thorough quality control, first performed in each batch of genotype data separately (EIRA batch one and two, SRQb batch one, two and three), then repeated after merging data. In particular, quality control steps included (i) filtering variants on missingness (>0.95), (ii) filtering samples on missingness (>0.98), (iii) filtering samples on mismatch between reported and inferred sex (F < 0.2 interpreted as female; F > 0.8 interpreted as male), (iv) filtering variants on missingness (>0.98), (v) filtering variants on differences in call rate between cases and controls (absolute difference > 0.02), (vi) filtering out monomorphic SNPs and (vii) filtering variants on Hardy-Weinberg equilibrium (HWE, p < 1e-10 in RA patients, p < 1e-6 in RA-free controls). In merged data, quality control additionally included (viii) filtering out closely related individuals (proportion identity-by-descent > 0.20) and (ix) filtering out individuals exhibiting non-homogeneous genetic ancestry (i.e., individuals more than six standard deviations from the principal component mean on any of the ten first principal components, passing over five iterations), wherein steps (viii-ix) were performed after performing linkage disequilibrium (LD) pruning (r^2^ > 0.20, in windows of 100 Mb, moving five SNPs at a time), and removing rare variants (minor allele frequency < 0.05) as well as variants not in HWE (p < 1e-3). Next, the post-quality-control data was harmonized against the reference panel data, i.e. the European subset of the Phase III 1000 Genomes data [11], using liftOver to realign the genotype data to GRCh37, and using R and PLINK to harmonize alleles of overlapping SNPs wherein duplicate variants and variants with ambiguous alleles were excluded. Pre-phasing was subsequently performed using SHAPEIT2 [12], with imputation of autosomal variants done with IMPUTE2 [13], resulting in the fully imputed genotype data under study.

A subset of individuals had further data on autoantibody-status, retrieved primarily as numerical measurements and dichotomized into positivity/negativity to reflect the clinical setting: in EIRA, anti-citrullinated peptide antibodies (ACPA) were determined by screening of baseline sera for IgG anti-CCP2 using CCPlus® ELISA (Euro-Diagnostica AB, Malmö, Sweden) according to the manufacturer’s instructions, with positivity defined as ≥25 arbitrary-units/ml; in SRQb, participants were screened for anti-CCP2 using the EliA^TM^ CCP assay (Thermo Fisher Scientific, Uppsala, Sweden) with positivity defined as >10 arbitrary-units/ml. In EIRA, cutoff for RF-positivity was chosen such that 2% of the controls were classified as positive; in SRQb, RF-status was reported by the treating clinician. In patients with missing data on RF and/or ACPA, autoantibody status was inferred from ICD-10 codes for the RA diagnosis as reported in SRQ.

**Data pre-processing and quality control**

Prior to analysis, non-genetic features were thoroughly processed. Firstly, among the medical history and drug prescription features, we excluded any variable with an observed prevalence < 0.01. Secondly, we assessed pairwise concordance across all medical history and drug prescription features, further excluding the feature with lowest observed variance from each pair exhibiting a proportion concordant > 0.95. Next, missing data were subsequently imputed using the full set of non-genetic features (i.e., demographics, clinical variables, medical- and drug prescription history data), using a random forest-approach as implemented in the `missForest` R package [14]. Finally, we used the fully imputed data construct additional compositive features (i.e., DAS28-ESR and DAS28-CRP), and standardized all continuous features to mean-zero and variance-one. Categorical variables were modeled as binary zero-one, with smoking modeled as never/former/current and educational attainment as > 9 years, 9-12 years and < 12 years, in line with existing conventions. Details on the full set of non-genetic features remaining after quality control, and their distributions across the two primary outcomes, are available in Table S2-S5.

Similarly, the fully imputed genotype data was processed through a stringent quality control across both individuals and genetic variants, comparable to that preceding imputation. In particular, samples were excluded based on the following criteria: (i) variant imputation INFO score < 0.70; (ii) sample mismatch between reported and inferred sex; (iii) sample missingness > 0.95; (iv) variant missingness > 0.95; (v) variant minor allele frequency < 0.01; (vi) variant deviation from HWE (p < 1e-6 in the full cohort); (vii) filtering out closely related samples (proportion identity-by-descent > 0.125); (viii) filtering out samples exhibiting non-homogeneous genetic ancestry. Here, the final two quality control steps, (vii) and (viii) were performed in a subset of SNPs where high LD SNPs had been withheld to avoid biases from highly correlated SNPs, as identified through a standard LD pruning. All quality control steps were carried out in PLINK. Results of the quality control procedure are presented in Table S7.

Among the individuals and variants remaining after quality control, two approaches to dimensionality reduction were employed. For the first approach, we used principal component analysis to transform the SNPs remaining after quality control into distinct principal components, each component used as an individual training feature. The number of principal components included into the training data was decided based on visual inspection of the resulting scree plots. For the second approach, we first extracted the subset of directly genotyped variants, i.e. excluded all imputed variants. We then used two rounds of LD pruning to obtain a set of near-independent variants, targeting variants in short-range LD during the first round (window of 1000 base pairs, r^2^ > 0.01) followed by a more lenient filtering targeting remaining variants in long-range LD (10,000 base pairs, r^2^ > 0.05). The subsequently remaining variants were included as individual features modeled at an additive scale, i.e. ranging from zero to two per the number of measured instances of the minor allele. Both the LD pruning and the principal component analysis was performed with PLINK.

Next, individual polygenic risk scores (PRSs) were constructed by an approach combining standard ‘clumping-and-thresholding’ [15], with principal component analysis [16]. Here, we first obtained independent GWAS summary statistic data from the UK Biobank for each of the phenotypes of interest (Table S6). Next, to identify a set of near-independent variants, we performed LD clumping (r^2^ > 0.10, across windows of 250kb) using the European sub-cohort of the 1000 Genomes Phase III data as reference panel [11], an approach which targets and excludes pairwise correlated variants while prioritizing the retention of highly associated SNPs. The clumped data was subsequently filtered through eleven p-value thresholds (p < [5 $\cdot$ 10^-10^, 1 $\cdot$ 10^-7^, 1 $\cdot$ 10^-6^, 1 $\cdot$ 10^-5^, 1 $\cdot$ 10^-4^, 1 $\cdot$ 10^.3^, 0.01, 0.05, 0.10, 0.50, 1.00$]$), resulting in eleven nested sets of variants, each of which was used to create a PRS. In line with Coombes et al., principal component analysis was then performed on the eleven PRSs [15], after which the first principal component was extracted and taken forward for analysis. Reference panel data was processed through a quality control procedure identical to that described for our fully imputed genotype data, prior to its use within LD clumping. Reference panel quality control and LD clumping was performed with PLINK, while the PRSs were constructed using R.

**Supervised learning parameter tuning**

All machine learning models except for logistic regression were taken through an inner cross-validation loop, with the purpose of identifying appropriate sets of hyperparameters that maximize the area under the curve (AUC). More specifically, we used ten-fold cross-validation, where 10% of data was kept as hold-out for evaluation, with the remaining 90% used to train the model for each set of hyperparameters. The set of hyperparameters achieving the highest AUC on the hold-out data was taken forward as the final model. Grid search was used for all three models.

For elastic net regression, we optimized the model for hyperparameters $\alpha\in\left[ 0,1 \right]$ – which regulates the regression model between ridge regression ($\alpha=0$) and LASSO ($\alpha=1$) – and $\lambda> 0$ – which regulates the shrinkage applied to features. For $\alpha$, we assessed a total of 23 values in [0, 0.001, 0.05, 0.10, …, 0.90, 0.95, 0.999, 1.00], 21 of which are in equidistant steps across the range of possible values, and two of which are borderline boundaries (i.e. $\alpha= 0.001$ and $\alpha= 0.999$) that are known to be able to deal with degeneracies caused by extreme correlations. For $\lambda$, we searched across a grid of 100 values between ${10}^{-4}$ and ${10}^{4}$, each value chosen by taking equidistant steps on the log-scale across the interval.

For random forest, we performed a grid search only for the mtry parameter, regulating the number of randomly selected features to assess within each decision tree of the forest, thus taking values in $\left[ 1,M \right]$, where *M* is the number of included features. A common choice known to perform well off-the-shelf is to set mtry to the square-root of *M*, and we therefore performed the grid search over 19 values in $\left[ 1,M \right]$ all equidistant from $\sqrt{M}$, i.e. ten values from $[1, \sqrt{M}]$ and ten values from [$\sqrt{M}, M]$, rounding all numbers down to obtain integers. Other parameters were kept fixed throughout, including the minimum size of a terminal node fixed at one, using the Gini-index as the evaluation metric for deciding tree splits, and training 1500 trees within each forest.

Lastly, for extreme gradient boosting (XGboost), five hyperparameters were trained, including the shrinkage parameter $\eta\in\left( 0,1 \right]$, and the number of sequential iterations *B ≥ 1*, which collectively control the learning rate, as well as the maximum tree depth ≥ 1, which controls the decision tree building, and the proportion of individuals and features randomly subsampled, $p_{r}\in\left( 0,1 \right]$ and $p_{c}\in\left( 0,1 \right]$. We evaluated the model for six values of *B*, with $B \in[50, 100, 500, 1000, 5000, 10000]$, and for 20 values of η, the first ten chosen to be equidistant on log-scale between 0.001 and 0.3, the other ten chosen to be equidistant on log-scale between 0.4 and 0.999, where mid-points of $\eta= 0.3$ and $\eta= 0.4$ are recommended values for off-the-shelf use. Seven values were assessed for the maximum tree depth, ranging over $[1, 3, 6, 10, 20, 50, {10}^{6}],$ the first corresponding to ‘stumps’, with the final value representing a tree with no maximum depth. Subsampling of individuals ranged over six values with $p_{r}\in[0.35, 0.50, 0.65, 0.80, 0.95, 1.00]$, while subsampling of features ranged over four with $p_{c}\in[0.25, 0.50, 0.75, 1.00]$. Other parameters include the $\gamma$ and the ‘minimum child weight’, which both add to the regularization of the tree by adding criteria that the tree needs to fulfill at each attempted split to commit to the proposed split, parameters which were here kept fixed at 0 for the former and 1 for the latter. The model was then evaluated for each of the 6 $\cdot$ 20 $\cdot$ 7 $\cdot$ 6 $\cdot$4 = 20,160 unique combinations, the best performing combination being taken forward as the final model.

**Alternative feature importance**

Supplementing the SHAP feature importance analysis, we additionally contrasted differences in distributions of non-genetic characteristics across individuals correctly classified with high probability. To facilitate explicit comparison of model probabilities, we first assessed model calibration in terms of ‘calibration-in-the-large’, ‘weak calibration’ and by visual inspection of the flexible calibration curves (‘moderate calibration’) [17], attempting recalibration upon detection of deviations using Platt scaling, Isotonic Regression and Beta Calibration. Here, numerical assessment of calibration quality indicated no issue for either of the two outcomes (Table S13), though visual inspection of the calibration plots suggested poor spread of probabilities. As such, we attempted to recalibrate probabilities, finding that Beta Calibration resulted in stable performance (Figure S2-S3).

Following recalibration, adjusted model probabilities were taken forward for classification with cut-offs based on those obtained by maximization of Youden’s index. Raw probabilities were binned based on deciles, extracting all individuals correctly classified with a corresponding probability in the top decile. To test for distributional differences between the two groups, we used standard t-tests for numerical variables and χ^2^ for categorical variables.

**Evaluation of previously published models trained on genetic data**

Feature sets were based on how they were described in the original publications, using the set of 61 features described by Lim et al. [18], and the set of 165 features described by Myasoedova et al. [19]. Non-genetic features were made to replicate those described in the original publications, with genetic variants identified on rs-number, where non-matching variants were replaced by proxy SNPs. Here, proxies were identified by targeting SNPs in LD with the index variant in windows of 1 Mb, based on common variants (minor allele frequency > 1%) in the European subsample of the 1000 Genomes reference panel [11], among the set of bi-allelic autosomal variants. Training and prediction were subsequently done using random forest, which was found to be efficient within both studies.

In replicating the set of training features employed by Lim et al. [18], age at diagnosis was retained from among our demographic features, and number of children at time of MTX initiation was computed by linkage to the Multi-Generation Register; data on morning stiffness, hemoglobin value and platelet count were unavailable in our register linkage and were thus not considered for training here. Of the 56 SNPs included by Lim et al., 39 were immediately available among the set of variants remaining after quality control, with 12 replaced by proxy SNPs, among which the average r^2^ was 0.72; the remaining five variants could not be replaced by proxies, three of which had none of their proxies available within the post-quality control data, and two which were not identified in the reference panel data, both of which had minor allele frequencies below 1% in the European subsample of the 1000 Genomes data. The outcome, attaining DAS28 < 2.6 within two years of treatment with MTX, was defined as having any visit within the two years following MTX treatment initiation, where a DAS28-ESR < 2.6 had been recorded.

Similarly, in replicating the set of training features employed by Myasoeodova et al. [19]. age at diagnosis, sex, baseline DAS28-ESR, RF and smoking status were retained from among our demographic and clinical features. Of the 160 SNPs included by Myasoedova et al., 137 were immediately available among the set of variants remaining after quality control, with 17 replaced by proxy SNPs, among which the average r^2^ was 0.77; the remaining six variants could not be replaced by proxies, two of which had none of their proxies available within the post-quality control data, and four which were not identified in the reference panel data. Of the latter, three were considered multi-allelic in the reference panel data and one was non-autosomal. Target outcome was primary response (i.e. achieving either EULAR good- or moderate response) at three months, where the visit closest to 90 days following MTX initiation in a window of 60-120 days, was employed.

**SUPPLEMENTARY TABLES**

**Table S1: Previously published literature on prediction of MTX treatment response.** *Results from a literature search conducted in the Spring of 2024, targeting English-language papers indexed through PubMed, on rheumatoid arthritis AND machine learning AND methotrexate, all as MeSH terms.*

| **Study** | **Data source** | **Design** | **Features** | **Sample size** | **Outcome** | **Model** | **Results** |
| --- | --- | --- | --- | --- | --- | --- | --- |
| Cherlin et al. (2018) | MATURA consortium (UK) | Early RA,  MTX in monotherapy | Genome-wide common genetic variants | 657 patients | Change in log(CRP), change in sqrt(SJC28), at three-six months | Lasso,  Ridge,  Elastic Net, Random Forest, Support Vector Regression,  Sparse Partial Least Squares Regression,  Neural Networks | AUC from 0.43-0.57 |
| Duong et al. (2022) | Four RCT-arms | MTX-naïve RA, randomized to MTX | Demographics,  clinical features | 775 patients | A latent class representation of DAS28-ESR  at 12 and 24 weeks | LASSO | AUC = 0.79 (0.74-0.84) |
| Duquesne et al. (2023) | ESPOIR (French) and Leiden-EAC (Dutch), with external validation from RCT-arm (tREACH) | RA,  MTX in monotherapy | Demographics, clinical features,  basic biomarkers | 732 treatment sequences,  external validation on 138 sequences | EULAR response (good + moderate),  at 9 (+-6) months | LightGBM | AUC = 0.73 (0.62-0.74), with external validation AUC = 0.72 (0.63 - 0.80) |
| Gehringer et al. (2024) | RAMS (UK), with external validation from NOR-DMARD (Norwegian) | MTX-naïve RA,  initiating MTX | Demographics.  clinical features | 1632 patients,  external validation on 1506 patients | Multinomial outcome of (i) DAS28 > 3.2 or non-persistent,  (ii) DAS28 <= 3.2 and (iii) discontinuation due to adverse events, at six months | Multinomial logistic regression | AUC = 0.72 (0.69 - 0.74) with external validation AUC = 0.68 (0.66 - 0.71). |
| Gosselt et al. (2021) | Two RCT-arms (tREACH and U-Act-Early) | RA, initiating MTX | Demographics, clinical features,  selected SNPs. | 355 patients | DAS28 <= 3.2,  at three months | Logistic regression | AUC = 0.77 (0.68 - 0.86) |
| Lim et al. (2022a) | Patients at Tan Tock Seng Hospital (Singapore) | RA, on MTX for at least three months | Demographics, clinical features,  haplotypes | 349 patients | DAS28 < 2.6,  within/at 2 years | Logistic Regression and Boosted Trees | AUC = 0.91,  with hold-out validation AUC = 0.83 |
| Lim et al. (2022b) | Patients at Tan Tock Seng Hospital (Singapore) | RA, on MTX for at least three months | Demographics, clinical features, SNPs | 349 patients | DAS28 < 2.6,  within/at 2 years | Neural Network  and Random Forest | AUC = 0.92,  with hold-out validation AUC = 0.83. |
| Myasoedova et al. (2022) | PAMERA consortium | MTX-naïve RA,  MTX in monotherapy | Demographics, clinical features, 160 SNPs found to be suggestive | 643 patients | EULAR response (good + moderate),  at 3 months | Random Forest | AUC = 0.83,  with hold-out validation AUC = 0.82 |
| Westerlind et al. (2021) | SRQ (Swedish) | Early MTX-naïve RA, MTX in monotherapy | Demographics, clinical features, medical history | 5475 patients | Persistence to MTX,  at one year | LASSO | AUC = 0.67 (0.62-0.71) |

**Table S2: Demographic features, within the study cohort.** *As measured at baseline, prior to imputation of missing data and standardization of features, with categorical variables represented by counts, and quantitative variables as medians. Missing observations were excluded from computations of these statistics.*

|  | **Overall** | **Persistence at one year** | | **Persistence at three years** | |
| --- | --- | --- | --- | --- | --- |
|  | *N = 2387* | *Non-persistent N =778* | *Persistent*  *N = 1604* | *Non-persistent*  *N = 1227* | *Persistent*  *N = 1075* |
| **Demographics** |  |  |  |  |  |
| *Female (%)* | 1621 (68%) | 556 (71%) | 1062 (66%) | 871 (71%) | 698 (65%) |
| *Age (IQR)* | 60 (19) | 57 (20) | 61 (17) | 57 (19) | 62 (16) |
| *Born in Sweden (%)* | 2137 (90%) | 691 (89%) | 1441 (90%) | 1085 (88%) | 974 (91%) |
| *First-degree relative with RA (%)* | 75 (3%) | 25 (3%) | 50 (3%) | 40 (3%) | 32 (3%) |
| **Educational level†** |  |  |  |  |  |
| *< 9 years (%)* | 517 (22%) | 163 (21%) | 353 (23%) | 250 (20%) | 255 (24%) |
| *9 – 12 years (%)* | 1094 (47%) | 349 (46%) | 745 (48%) | 576 (47%) | 509 (47%) |
| *≥ 12 years (%)* | 717 (31%) | 247 (33%) | 469 (30%) | 400 (33%) | 308 (29%) |
| **Smoking status‡** |  |  |  |  |  |
| *Never (%)* | 268 (37%) | 64 (28%) | 204 (41%) | 120 (36%) | 130 (38%) |
| *Previous (%)* | 330 (45%) | 111 (49%) | 219 (44%) | 152 (45%) | 152 (45%) |
| *Current (%)* | 129 (18%) | 52 (23%) | 76 (15%) | 66 (20%) | 56 (17%) |
| **Healthcare utilization** |  |  |  |  |  |
| *Days hospitalized (IQR; min-max)* | 0 (0; 0-58) | 0 (0; 0-56) | 0 (0; 0-58) | 0 (0; 0-56) | 0 (0; 0-45) |
| *Days on disability pension (IQR; min-max)* | 0 (0; 0-365) | 0 (0; 0-365) | 0 (0; 0-365) | 0 (0; 0-365) | 0 (0; 0-365) |
| *Days on sick leave (IQR; min-max)* | 0 (0; 0-365) | 0 (9; 0-365) | 0 (0; 0-365) | 0 (2; 0-365) | 0 (0; 0-365) |
| *Prescription expenditures, in SEK (IQR)* | 19401 (3228) | 1942 (3155) | 1946 (3250) | 1880 (2977) | 1983 (3367) |
| †: 59 (2%) individuals had missing data on education level; ‡: 1660 (70%) of these had missing data on smoking status. | | | | | |

**Table S3: Clinical features, within the study cohort.** *As measured at baseline, prior to imputation of missing data and standardization of features, with categorical variables represented by counts, and quantitative variables as medians. Missing observations were excluded from computations of these statistics.*

|  | **Overall** | **Persistence at one year** | | **Persistence at three years** | |
| --- | --- | --- | --- | --- | --- |
|  | *N = 2387* | *Non-persistent N = 778* | *Persistent*  *N = 1604* | *Non-persistent N =1227* | *Persistent*  *N = 1075* |
| **Disease characteristics** |  |  |  |  |  |
| *Symptom duration, in months (IQR)* | 5 (4) | 5 (5) | 5 (4) | 5 (4) | 5 (4) |
| *Seropositive^a^ (%)* | 1695 (76%) | 563 (76%) | 1128 (75%) | 903 (77%) | 736 (73%) |
| **Disease activity components** |  |  |  |  |  |
| *Swollen joint count, 28 joints^b^ (IQR)* | 7 (7) | 7 (6) | 7 (7) | 7 (7) | 7 (7) |
| *Tender joint count, 28 joints^c^ (IQR)* | 6 (8) | 7 (8) | 6 (7) | 7 (8) | 6 (7) |
| *Erythrocyte sedimentation rate^d^ (IQR)* | 26 (29) | 27 (29) | 26 (28) | 26 (30) | 25 (27) |
| *C-reactive protein levels^e^ (IQR)* | 11 (23) | 13 (27) | 10 (20) | 11 (24) | 11 (20) |
| *Patient Global Health^f^ (IQR)* | 50 (39) | 55 (37) | 48 (38) | 54 (37) | 47 (38) |
| **Additional clinical measurements** |  |  |  |  |  |
| *Health Assessment Questionnaire^g^ (IQR)* | 1 (0.75) | 1.13 (0.75) | 1 (0.88) | 1 (0.8) | 1 (0.8) |
| *Pain^h^ (IQR)* | 54 (39) | 60 (38) | 51 (37) | 56 (40) | 50 (39) |
| **Additional therapies** |  |  |  |  |  |
| *Corticosteroids^i^ (%)* | 1528 (70%) | 482 (69%) | 1043 (71%) | 783 (70%) | 677 (69%) |
| *Non-steroidal anti-inflammatory drugs^i^ (%)* | 548 (25%) | 186 (27%) | 360 (24%) | 280 (25%) | 253 (26%) |
| *a:* 146 (6%) of these had missing data on seropositivity;  *b*: 266 (11%) of these had missing data on swollen joint count;  *c*: 265 (11%) of these had missing data on tender joint count;  *d:* 305 (13%) of these had missing data on erythrocyte sedimentation rate;  *e:* 261 (11%) of these had missing data on C-reactive protein levels;  *f:* 361 (15%) of these had missing data on patient global health;  *g:* 409 (17%) of these had missing data on Health Assessment Questionnaire;  *h*: 361 (15%) of these had missing data on pain;  *i:* 211 (8%) of these had missing data on corticosteroid and non-steroidal anti-inflammatory drug treatment. | | | | | |

**Table S4: Medical history features, within the study cohort.** *As described by the occurrence of ICD-10 codes from the five years preceding the first prescription of MTX, remaining after filtering on low variance and high concordance.*

|  | **Overall** | **Persistence at one year** | | **Persistence at three years** | |
| --- | --- | --- | --- | --- | --- |
|  | *N = 2387* | *Non-persistent*  *N = 778* | *Persistent*  *N = 1604* | *Non-persistent N = 1227* | *Persistent*  *N = 1075* |
| **Diseases of the blood and blood-forming organs and certain disorders involving the immune mechanism (D00-D99)** |  |  |  |  |  |
| *Benign neoplasms (D10-D36)* | 163 (7%) | 101 (6%) | 62 (8%) | 71 (7%) | 84 (7%) |
| *Neoplasms of uncertain or unspecified nature (D37-D48)* | 108 (5%) | 76 (5%) | 32 (4%) | 52 (5%) | 48 (4%) |
| **Diseases of the nervous system (G00-G99)** |  |  |  |  |  |
| *Episodic and paroxysmal disorders (G40-G47)* | 95 (4%) | 64 (4%) | 31 (4%) | 48 (4%) | 45 (4%) |
| *Nerve, nerve root and plexus disorders (G50-G59)* | 110 (5%) | 71 (4%) | 39 (5%) | 42 (4%) | 63 (5%) |
| **Diseases of the eye, adnexa, ear and mastoid process (H00-H59)** |  |  |  |  |  |
| *Disorders of eyelid, lacrimal system and orbit (H00-H06)* | 91 (4%) | 54 (3%) | 37 (5%) | 41 (4%) | 42 (3%) |
| *Disorders of sclera, cornea, iris and ciliary body (H15-H22)* | 70 (3%) | 42 (3%) | 27 (3%) | 27 (3%) | 37 (3%) |
| *Disorders of lens (H25-H28)* | 177 (7%) | 116 (7%) | 61 (8%) | 76 (7%) | 84 (7%) |
| *Disorders of choroid and retina (H30-H36)* | 82 (3%) | 60 (4%) | 22 (3%) | 39 (4%) | 36 (3%) |
| *Other disorders of the ear (H90-H95)* | 89 (4%) | 61 (4%) | 28 (4%) | 49 (5%) | 37 (3%) |
| **Diseases of the circulatory system (I00-I99)** |  |  |  |  |  |
| *Hypertensive diseases (I10-I15)* | 74 (3%) | 54 (3%) | 20 (3%) | 41 (4%) | 33 (3%) |
| *Other forms of heart disease (I30-I52)* | 105 (4%) | 79 (5%) | 26 (3%) | 58 (5%) | 40 (3%) |
| *Diseases of veins, lymphatic vessels and lymph nodes, not elsewhere classified (I80-I89)* | 88 (4%) | 63 (4%) | 25 (3%) | 43 (4%) | 42 (3%) |
| **Diseases of the respiratory system (J00-J99)** |  |  |  |  |  |
| *Other diseases of upper respiratory tract (J30-J39)* | 110 (5%) | 75 (5%) | 35 (4%) | 43 (4%) | 61 (5%) |
| **Diseases of the digestive system (K00-K95)** |  |  |  |  |  |
| *Diseases of esophagus, stomach and duodenum (K20-K31)* | 80 (3%) | 46 (3%) | 34 (4%) | 34 (3%) | 43 (4%) |
| *Hernia (K40-K46)* | 63 (3%) | 46 (3%) | 17 (2%) | 32 (3%) | 28 (2%) |
| *Other diseases of intestines (K55-K64)* | 103 (4%) | 79 (5%) | 24 (3%) | 51 (5%) | 43 (4%) |
| **Diseases of the skin and subcutaneous tissue (L00-L99)** |  |  |  |  |  |
| *Dermatitis and eczema (L20-L30)* | 94 (4%) | 60 (4%) | 34 (4%) | 38 (4%) | 47 (4%) |
| *Radiation-related disorders of the skin and subcutaneous tissue (L55-L59)* | 67 (3%) | 50 (3%) | 17 (2%) | 36 (3%) | 26 (2%) |
| *Other disorders of the skin and subcutaneous tissue (L80-L99)* | 109 (5%) | 75 (5%) | 33 (4%) | 47 (4%) | 53 (4%) |
| **Diseases of the musculoskeletal system and connective tissue (M00-M99)** |  |  |  |  |  |
| *Inflammatory polyarthropathies (M05-M14)* | 2298 (96%) | 1550 (97%) | 743 (96%) | 1034 (96%) | 1181 (96%) |
| *Osteoarthritis (M15-M19)* | 267 (11%) | 186 (12%) | 81 (10%) | 125 (12%) | 131 (11%) |
| *Other joint disorders (M20-M25)* | 419 (18%) | 254 (16%) | 165 (21%) | 163 (15%) | 240 (20%) |
| *Other dorsopathies (M50-M54)* | 113 (5%) | 75 (5%) | 38 (5%) | 48 (4%) | 61 (5%) |
| *Disorders of synovium and tendon (M65-M68)* | 119 (5%) | 78 (5%) | 41 (5%) | 44 (4%) | 70 (6%) |
| *Other soft tissue disorders (M70-M79)* | 481 (20%) | 309 (19%) | 172 (22%) | 192 (18%) | 270 (22%) |
| **Diseases of the genitourinary system (N00-N99)** |  |  |  |  |  |
| *Other diseases of the urinary system (N30-N39)* | 93 (4%) | 59 (4%) | 34 (4%) | 41 (4%) | 49 (4%) |
| *Noninflammatory disorders of female genital tract (N80-N98)* | 280 (12%) | 165 (10%) | 115 (15%) | 112 (10%) | 151 (12%) |
| **Symptoms, signs and abnormal clinical and laboratory findings, not elsewhere classified (R00-R99)** |  |  |  |  |  |
| *Symptoms and signs involving the circulatory and respiratory systems (R00-R09)* | 239 (10%) | 162 (10%) | 77 (10%) | 114 (11%) | 112 (9%) |
| *Symptoms and signs involving the digestive system and abdomen (R10-R19)* | 172 (7%) | 95 (6%) | 76 (10%) | 70 (7%) | 93 (8%) |
| *Symptoms and signs involving the skin and subcutaneous tissue (R20-R23)* | 91 (4%) | 58 (4%) | 33 (4%) | 39 (4%) | 48 (4%) |
| *Symptoms and signs involving cognition, perception, emotional state and behavior (R40-R46)* | 59 (2%) | 42 (3%) | 17 (2%) | 31 (3%) | 25 (2%) |
| *General symptoms and signs (R50-R69)* | 203 (9%) | 131 (8%) | 72 (9%) | 92 (9%) | 105 (9%) |
| **Injury, poisoning and certain other consequences of external causes (S00-S99)** |  |  |  |  |  |
| *Injuries to the wrist, hand and fingers (S60-S69)* | 84 (4%) | 58 (4%) | 26 (3%) | 43 (4%) | 39 (3%) |
| *Injuries to the knee and lower leg (S80-S89)* | 94 (4%) | 57 (4%) | 35 (4%) | 37 (3%) | 50 (4%) |
| **Factors influencing health status and contact with health services (Z00-Z99)** |  |  |  |  |  |
| *Encountering health services for examinations (Z00-Z13)* | 642 (27%) | 422 (26%) | 218 (28%) | 280 (26%) | 330 (27%) |
| *Encountering health services in circumstances related to reproduction (Z30-Z39)* | 82 (3%) | 51 (3%) | 31 (4%) | 28 (3%) | 50 (4%) |
| *Encounters for other specific health care (Z40-Z54)* | 100 (4%) | 67 (4%) | 33 (4%) | 50 (5%) | 41 (3%) |
| *Encountering health services in other circumstances (Z70-Z76)* | 97 (4%) | 68 (4%) | 29 (4%) | 37 (3%) | 58 (5%) |
| *Potential health hazards related to family and personal history and certain conditions influencing health status (Z80-Z99)* | 161 (7%) | 99 (6%) | 62 (8%) | 65 (6%) | 82 (7%) |

**Table S5: Drug prescription features, within the study cohort.** *As described by the occurrence of four-character ATC-codes from the year immediately preceding the first prescription of MTX, remaining after filtering on low variance and high concordance.*

|  | **Overall** | **Persistence at one year** | | **Persistence at three years** | |
| --- | --- | --- | --- | --- | --- |
|  | *N = 2387* | *Non-persistent N = 778* | *Persistent*  *N = 1604* | *Non-persistent N = 1075* | *Persistent*  *N = 1227* |
| **Alimentary tract and metabolism (A)** |  |  |  |  |  |
| *Stomatological preparations (A01A)* | 124 (5%) | 78 (5%) | 46 (6%) | 55 (5%) | 65 (5%) |
| *Antacids (A02B)* | 812 (34%) | 521 (32%) | 290 (37%) | 351 (33%) | 422 (34%) |
| *Drugs for constipation (A06A)* | 205 (9%) | 145 (9%) | 60 (8%) | 100 (9%) | 92 (7%) |
| *Blood glucose lowering drugs, excl. insulins (A10B)* | 101 (4%) | 76 (5%) | 25 (3%) | 59 (5%) | 38 (3%) |
| *Calcium (A12A)* | 914 (38%) | 625 (39%) | 288 (37%) | 419 (39%) | 454 (37%) |
| **Blood and blood forming organs (B)** |  |  |  |  |  |
| *Antithrombotic agents (B01A)* | 365 (15%) | 253 (16%) | 110 (14%) | 189 (18%) | 150 (12%) |
| *Iron preparations (B03A)* | 82 (3%) | 56 (3%) | 26 (3%) | 39 (4%) | 38 (3%) |
| **Cardiovascular system (C)** |  |  |  |  |  |
| *Low-ceiling diuretics, thiazides (C03A)* | 89 (4%) | 68 (4%) | 21 (3%) | 54 (5%) | 31 (3%) |
| *High-ceiling diuretics (C03C)* | 115 (5%) | 78 (5%) | 37 (5%) | 61 (6%) | 49 (4%) |
| *Beta blocking agents (C07A)* | 408 (17%) | 294 (18%) | 111 (14%) | 208 (19%) | 178 (15%) |
| *Selective calcium channel blockers with mainly vascular effects (C08C)* | 285 (12%) | 196 (12%) | 87 (11%) | 145 (13%) | 125 (10%) |
| *Ace inhibitors, plain (C09A)* | 255 (11%) | 191 (12%) | 60 (8%) | 130 (12%) | 106 (9%) |
| *Angiotensin II receptor blockers (ARBs), plain (C09C)* | 210 (9%) | 150 (9%) | 60 (8%) | 100 (9%) | 101 (8%) |
| *Angiotensin II receptor blockers (ARBs), combinations (C09D)* | 113 (5%) | 77 (5%) | 36 (5%) | 60 (6%) | 50 (4%) |
| *Lipid modifying agents, plain (C10A)* | 381 (16%) | 278 (17%) | 101 (13%) | 195 (18%) | 167 (14%) |
| **Dermatologicals (D)** |  |  |  |  |  |
| *Antifungals for topical use (D01A)* | 96 (4%) | 61 (4%) | 35 (4%) | 40 (4%) | 53 (4%) |
| *Emollients and protectives (D02A)* | 159 (7%) | 110 (7%) | 49 (6%) | 73 (7%) | 76 (6%) |
| *Corticosteroids, plain (D07A)* | 244 (10%) | 159 (10%) | 84 (11%) | 112 (10%) | 124 (10%) |
| **Genito urinary system and sex hormones (G)** |  |  |  |  |  |
| *Hormonal contraceptives for systemic use (G03A)* | 133 (6%) | 78 (5%) | 55 (7%) | 48 (4%) | 83 (7%) |
| *Estrogens (G03C)* | 211 (9%) | 138 (9%) | 72 (9%) | 105 (10%) | 100 (8%) |
| *Urologicals (G04B)* | 98 (4%) | 63 (4%) | 34 (4%) | 41 (4%) | 46 (4%) |
| **Systemic hormonal preparations, excl. sex hormones and insulins (H)** |  |  |  |  |  |
| *Corticosteroids for systemic use, plain (H02A)* | 1516 (64%) | 1011 (63%) | 503 (65%) | 666 (62%) | 789 (64%) |
| *Thyroid preparations (H03A)* | 253 (11%) | 166 (10%) | 87 (11%) | 105 (10%) | 139 (11%) |
| **Antiinfectives for systemic use (J)** |  |  |  |  |  |
| *Tetracyclines (J01A)* | 223 (9%) | 132 (8%) | 91 (12%) | 82 (8%) | 131 (11%) |
| *Beta-lactam antibactericals, penicillins (J01C)* | 482 (20%) | 317 (20%) | 164 (21%) | 209 (19%) | 258 (21%) |
| *Macrolides, lincosamides and streptogramins (J01F)* | 79 (3%) | 49 (3%) | 29 (4%) | 31 (3%) | 45 (4%) |
| *Quinolone antibacterials (J01M)* | 67 (3%) | 47 (3%) | 20 (3%) | 32 (3%) | 33 (3%) |
| *Other antibacterials (J01X)* | 73 (3%) | 48 (3%) | 25 (3%) | 29 (3%) | 40 (3%) |
| **Antineoplastic and immunomodulating agents (L)** |  |  |  |  |  |
| *Immunosuppressant (L04A)* | 1378 (58%) | 927 (58%) | 449 (58%) | 627 (58%) | 698 (57%) |
| **Musculo-skeletal system (M)** |  |  |  |  |  |
| *Antiinflammatory and antirheumatic products, non-steroids (M01A)* | 1963 (82%) | 1304 (81%) | 656 (84%) | 865 (80%) | 1034 (84%) |
| *Topical products for joint and muscular pain (M02A)* | 110 (5%) | 77 (5%) | 33 (4%) | 55 (5%) | 52 (4%) |
| *Muscle relaxants, centrally acting agents (M03B)* | 72 (3%) | 50 (3%) | 22 (3%) | 31 (3%) | 38 (3%) |
| *Drugs affecting bone structure and mineralization (M05B)* | 109 (5%) | 69 (4%) | 39 (5%) | 52 (5%) | 48 (4%) |
| **Nervous system (N)** |  |  |  |  |  |
| *Opioids (N02A)* | 702 (29%) | 433 (27%) | 269 (35%) | 271 (25%) | 403 (33%) |
| *Other analgesics and antipyretics (N02B)* | 1061 (44%) | 685 (43%) | 376 (48%) | 460 (43%) | 565 (46%) |
| *Anxiolytics (N05B)* | 139 (6%) | 89 (6%) | 50 (6%) | 64 (6%) | 70 (6%) |
| *Hypnotics and sedatives (N05C)* | 320 (13%) | 212 (13%) | 107 (14%) | 148 (14%) | 157 (13%) |
| *Antidepressants (N06A)* | 272 (11%) | 178 (11%) | 94 (12%) | 111 (10%) | 149 (12%) |
| **Respiratory system (R)** |  |  |  |  |  |
| *Decongestants and other nasal preparations for topical use (R01A)* | 248 (10%) | 159 (10%) | 89 (11%) | 113 (11%) | 128 (10%) |
| *Adrenergics, inhalants (R03A)* | 238 (10%) | 156 (10%) | 82 (11%) | 101 (9%) | 123 (10%) |
| *Other drugs for obstructive airway diseases, inhalants (R03B)* | 170 (7%) | 115 (7%) | 55 (7%) | 73 (7%) | 85 (7%) |
| *Expectorants, excl. combinations with cough suppressants (R05C)* | 220 (9%) | 143 (9%) | 77 (10%) | 89 (8%) | 120 (10%) |
| *Cough suppressants and expectorants, combinations (R05F)* | 164 (7%) | 94 (6%) | 70 (9%) | 58 (5%) | 99 (8%) |
| Antihistamines for systemic use (R06A) | 204 (9%) | 123 (8%) | 81 (10%) | 79 (7%) | 119 (10%) |
| **Sensory organs (S)** |  |  |  |  |  |
| *Corticosteroids and antiinfectives in combination (S03C)* | 73 (3%) | 41 (3%) | 32 (4%) | 26 (2%) | 43 (4%) |

**Table S6: Quality control procedure.** *Summary of the samples and variants removed during the quality control procedure for the genotype data in the study cohort.*

|  | Individuals | Variants |
| --- | --- | --- |
| Raw Data | **2432** | **20,560,897** |
| *SNP imputation quality ≥ 0.70* | 2432 | 14,291,832 |
| *Reported sex matching inferred sex, and non-ambiguous inferred sex* | 2413 | 14,291,832 |
| *Individual call rate ≥ 0.95* | 2413 | 14,291,832 |
| *SNP call rate ≥ 0.95* | 2413 | 12,744,286 |
| *Minor allele frequency ≥ 0.01* | 2413 | 7,652,931 |
| *No deviation from Hardy-Weinberg equilibrium (p ≥ 1e-6)* | 2413 | 7,652,909 |
| *Pairwise relatedness < 0.125* | 2388 | 7,652,909 |
| *Homogenous genetic ancestry* | 2387 | 7,652,909 |
| Final Data | **2387** | **7,652,909** |

**Table S7: Phenotypes for which UK Biobank GWAS data were extracted and employed in the creation of PRSs.**

| **TARGET PHENOTYPE** | **UK BIOBANK PHENOTYPE** | **NUMBER OF CASES** |
| --- | --- | --- |
| Asthma | Diagnoses - main ICD10: J45 Asthma | 1693 (0.47%) |
| Bipolar Disorder | Diagnoses - main ICD10: F31 Bipolar affective disorder | 371 (0.10%) |
| Body Mass Index | Body mass index (BMI) | 359,983 |
| Chronic Obstructive Pulmonary Disease | Diagnoses - main ICD10: J44 Other chronic obstructive pulmonary disease | 1531 (0.42%) |
| C-reactive Protein | C-reactive protein (quantile) | 343,524 |
| Crohn’s Disease | Diagnoses - main ICD10: K50 Crohn's disease [regional enteritis] | 968 (0.27%) |
| Depression | Diagnoses - main ICD10: F33 Recurrent depressive disorder | 293 (0.08%) |
| Diabetes, Type I | Type 1 diabetes | 583 (0.16%) |
| Diabetes, Type II | Type 2 diabetes | 888 (0.25%) |
| Hypertension | Diagnoses - main ICD10: I10 Essential (primary) hypertension | 787 (0.22%) |
| Hyperthyroidism | Diagnoses - main ICD10: E05 Thyrotoxicosis [hyperthyroidism] | 392 (0.11%) |
| Rheumatoid Arthritis | Rheumatoid arthritis | 1605 (0.44%) |
| Schizophrenia | Diagnoses - main ICD10: F20 Schizophrenia | 198 (0.05%) |
| Smoking | Tobacco smoking: Smokes on most or all days | 2124 (2.32%) |
| Ulcer, Gastric | Diagnoses - main ICD10: K25 Gastric ulcer | 1834 (0.51%) |
| Ulcer, Duodenal | Diagnoses - main ICD10: K26 Duodenal ulcer | 1291 (0.36%) |
| Ulcerative Colitis | Diagnoses - main ICD10: K51 Ulcerative colitis | 2143 (0.59%) |

**Table S8: Reclassification statistics**. *Net reclassification and total reclassification for both persistence at one year and persistence at three years, assessed for each of the four prediction algorithms, when adding each set of genetic features to the non-genetic characteristics.*

|  | *Reclassification after including PRS* | *Reclassification after including PRS and principal components* | *Reclassification after including PRS and near-independent genotyped variants* |
| --- | --- | --- | --- |
| **Persistence at one year** |  |  |  |
| *Logistic regression, total (net)* | 0.05 (0.00) | 0.06 (0.00) |  |
| *Elastic net regression, total (net)* | 0.01 (0.00) | 0.01 (-0.01) | 0.01 (-0.01) |
| *Random forest, total (net)* | 0.06 (-0.04) | 0.06 (-0.03) | 0.07 (-0.05) |
| *XGBoost, total (net)* | 0.05 (0.01) | 0.08 (-0.01) | 0.15 (-0.04) |
| **Persistence at three years** |  |  |  |
| *Logistic regression, total (net)* | 0.08 (-0.01) | 0.08 (-0.01) |  |
| *Elastic net regression, total (net)* | 0.06 (-0.02) | 0.05 (-0.02) | 0.22 (-0.08) |
| *Random forest, total (net)* | 0.17 (-0.02) | 0.18 (-0.02) | 0.24 (-0.02) |
| *XGBoost, total (net)* | 0.17 (-0.02) | 0.19 (-0.02) | 0.33 (-0.07) |

**Table S9: Model prediction quality in seropositive and seronegative patients.** *Model quality in predicting persistence to treatment with MTX at one and three years after treatment start, respectively, for four distinct models, trained on four distinct sets of training features, in patients with seropositive and seronegative RA, respectively*. *Estimates of AUC are averaged across the cross-validation folds; confidence intervals are computed through 2000 stratified bootstrap replicates.*

|  | *Non-genetic characteristics* | *Non-genetic characteristics,*  *and PRS* | *Non-genetic characteristics, PRS and principal components* | *Non-genetic characteristics, PRS, and near-independent genotyped variants* |
| --- | --- | --- | --- | --- |
| **SEROPOSITIVE PATIENTS, N = 1695** |  |  |  |  |
| **Persistence at one year,**  N = 1128 (67%) |  |  |  |  |
| *Logistic regression, AUC (95% CI)* | 0.602 (0.537 - 0.666) | 0.603 (0.538 - 0.668) | 0.602 (0.537 - 0.666) | - |
| *Elastic net regression, AUC (95% CI)* | 0.631 (0.568 - 0.694) | 0.630 (0.566 - 0.693) | 0.631 (0.567 - 0.694) | 0.565 (0.500 – 0.630) |
| *Random forest, AUC (95% CI)* | 0.608 (0.544 - 0.672) | 0.602 (0.538 - 0.666) | 0.605 (0.541 - 0.670) | 0.601 (0.536 - 0.666) |
| *XGBoost, AUC (95% CI)* | 0.616 (0.552 - 0.680) | 0.610 (0.545 - 0.675) | 0.599 (0.535 - 0.663) | 0.525 (0.459 – 0.591) |
| **Persistence at three years,**  N = 736 (43%) |  |  |  |  |
| *Logistic regression, AUC (95% CI)* | 0.610 (0.549 - 0.671) | 0.611 (0.550 - 0.672) | 0.607 (0.546 - 0.668) | - |
| *Elastic net regression, AUC (95% CI)* | 0.635 (0.575 - 0.695) | 0.642 (0.583 - 0.702) | 0.639 (0.579 - 0.699) | 0.559 (0.620 – 0.680) |
| *Random forest, AUC (95% CI)* | 0.630 (0.569 - 0.690) | 0.636 (0.575 - 0.696) | 0.634 (0.573 - 0.694) | 0.617 (0.556 - 0.677) |
| *XGBoost, AUC (95% CI)* | 0.627 (0.567 - 0.687) | 0.608 (0.548 - 0.669) | 0.624 (0.563 - 0.687) | 0.566 (0.504 – 0.628) |
| **SERONEGATIVE PATIENTS, N = 546** |  |  |  |  |
| **Persistence at one year,**  N = 367 (67%) |  |  |  |  |
| *Logistic regression, AUC (95% CI)* | 0.516 (0.397 - 0.635) | 0.517 (0.399 - 0.636) | 0.500 (0.381 - 0.619) | - |
| *Elastic net regression, AUC (95% CI)* | 0.630 (0.521 - 0.739) | 0.632 (0.523 - 0.741) | 0.629 (0.518 - 0.739) | 0.626 (0.512 – 0.740) |
| *Random forest, AUC (95% CI)* | 0.627 (0.511 - 0.742) | 0.633 (0.522 - 0.745) | 0.637 (0.525 - 0.748) | 0.572 (0.456 - 0.688) |
| *XGBoost, AUC (95% CI)* | 0.600 (0.485 - 0.716) | 0.602 (0.489 - 0.716) | 0.643 (0.534 - 0.752) | 0.593 (0.475 – 0.711) |
| **Persistence at three years,**  N = 270 (49%) |  |  |  |  |
| *Logistic regression, AUC (95% CI)* | 0.562 (0.452 - 0.672) | 0.544 (0.433 - 0.656) | 0.566 (0.455 - 0.677) | - |
| *Elastic net regression, AUC (95% CI)* | 0.597 (0.489 - 0.706) | 0.593 (0.485 - 0.701) | 0.592 (0.484 - 0.700) | 0.525 (0.435 – 0.615) |
| *Random forest, AUC (95% CI)* | 0.609 (0.502 - 0.716) | 0.591 (0.482 - 0.699) | 0.566 (0.457 - 0.675) | 0.548 (0.437 - 0.660) |
| *XGBoost, AUC (95% CI)* | 0.592 (0.483 - 0.701) | 0.559 (0.449 - 0.670) | 0.539 (0.428 - 0.650) | 0.549 (0.439 – 0.659) |

**Table S10: Model prediction quality towards alternative treatment outcomes.** *Model quality in predicting alternative MTX treatment outcomes for four distinct models, trained on four distinct sets of training features*. *Estimates of AUC are averaged across the cross-validation folds; confidence intervals are computed through 2000 stratified bootstrap replicates.*

|  | *Non-genetic characteristics* | *Non-genetic characteristics,*  *and PRS* | *Non-genetic characteristics, PRS and principal components* | *Non-genetic characteristics, PRS, and near-independent genotyped variants* |
| --- | --- | --- | --- | --- |
| **EULAR primary response, six months,**  N = 744 (51%) |  |  |  |  |
| *Logistic regression, AUC (95% CI)* | 0.619 (0.554 - 0.683) | 0.611 (0.546 - 0.676) | 0.609 (0.544 - 0.674) | - |
| *Elastic net regression, AUC (95% CI)* | 0.596 (0.531 - 0.662) | 0.608 (0.543 - 0.672) | 0.610 (0.545 - 0.675) | 0.531 (0.464 - 0.597) |
| *Random forest, AUC (95% CI)* | 0.663 (0.601 - 0.725) | 0.666 (0.604 - 0.728) | 0.659 (0.597 - 0.722) | 0.636 (0.573 - 0.700) |
| *XGBoost, AUC (95% CI)* | 0.651 (0.588 - 0.714) | 0.647 (0.584 - 0.710) | 0.637 (0.574 - 0.701) | 0.535 (0.469 - 0.602) |
| **DAS28-remission, one year,**  N = 825 (44%) |  |  |  |  |
| *Logistic regression, AUC (95% CI)* | 0.635 (0.578 - 0.691) | 0.637 (0.580 - 0.693) | 0.631 (0.575 - 0.688) | - |
| *Elastic net regression, AUC (95% CI)* | 0.653 (0.598 - 0.709) | 0.658 (0.602 - 0.713) | 0.659 (0.604 - 0.714) | 0.645 (0.588 - 0.701) |
| *Random forest, AUC (95% CI)* | 0.660 (0.604 - 0.715) | 0.663 (0.608 - 0.719) | 0.658 (0.603 - 0.714) | 0.638 (0.582 - 0.695) |
| *XGBoost, AUC (95% CI)* | 0.647 (0.591 - 0.703) | 0.649 (0.593 - 0.705) | 0.644 (0.588 - 0.700) | 0.606 (0.549 - 0.664) |
| **Remission at six months, and retention of MTX at one year,**  N = 674 (43%) |  |  |  |  |
| *Logistic regression, AUC (95% CI)* | 0.625 (0.563 - 0.687) | 0.628 (0.566 - 0.690) | 0.621 (0.559 - 0.683) | - |
| *Elastic net regression, AUC (95% CI)* | 0.639 (0.578 - 0.701) | 0.639 (0.577 - 0.700) | 0.638 (0.576 - 0.699) | 0.623 (0.560 - 0.685) |
| *Random forest, AUC (95% CI)* | 0.648 (0.587 – 0.701) | 0.664 (0.604 – 0.724) | 0.661 (0.600 – 0.721) | 0.647 (0.586 – 0.708) |
| *XGBoost, AUC (95% CI)* | 0.639 (0.577 - 0.701) | 0.640 (0.579 - 0.701) | 0.637 (0.576 - 0.699) | 0.580 (0.517 - 0.644) |
| **Remission at six months, and retention of MTX at three years,**  N = 587 (38%) |  |  |  |  |
| *Logistic regression, AUC (95% CI)* | 0.625 (0.562 - 0.689) | 0.622 (0.558 - 0.685) | 0.615 (0.551 - 0.679) | - |
| *Elastic net regression, AUC (95% CI)* | 0.627 (0.564 - 0.691) | 0.629 (0.566 - 0.693) | 0.626 (0.562 - 0.690) | 0.628 (0.564 - 0.692) |
| *Random forest, AUC (95% CI)* | 0.638 (0.574 – 0.701) | 0.648 (0.585 – 0.712) | 0.643 (0.579 – 0.706) | 0.639 (0.575 – 0.703) |
| *XGBoost, AUC (95% CI)* | 0.620 (0.556 - 0.684) | 0.626 (0.562 - 0.690) | 0.624 (0.560 - 0.688) | 0.582 (0.516 - 0.648) |
| **Discontinuation of MTX, one year,**  N = 277 (12%) |  |  |  |  |
| *Logistic regression, AUC (95% CI)* | 0.531 (0.449 - 0.614) | 0.520 (0.436 - 0.603) | 0.525 (0.443 - 0.607) | - |
| *Elastic net regression, AUC (95% CI)* | 0.513 (0.429 - 0.597) | 0.505 (0.422 - 0.588) | 0.507 (0.422 - 0.591) | 0.534 (0.454 - 0.614) |
| *Random forest, AUC (95% CI)* | 0.500 (0.417 – 0.584) | 0.516 (0.434 – 0.598) | 0.525 (0.443 – 0.607) | 0.543 (0.462 – 0.624) |
| *XGBoost, AUC (95% CI)* | 0.523 (0.441 - 0.605) | 0.512 (0.428 - 0.596) | 0.547 (0.467 - 0.628) | 0.550 (0.470 - 0.630) |
| **Discontinuation of MTX, three years,**  N = 512 (22%) |  |  |  |  |
| *Logistic regression, AUC (95% CI)* | 0.557 (0.493 - 0.621) | 0.557 (0.493 - 0.620) | 0.544 (0.481 - 0.608) | - |
| *Elastic net regression, AUC (95% CI)* | 0.568 (0.505 - 0.631) | 0.570 (0.507 - 0.633) | 0.576 (0.514 - 0.639) | 0.525 (0.462 - 0.588) |
| *Random forest, AUC (95% CI)* | 0.572 (0.507 – 0.637) | 0.557 (0.492 – 0.623) | 0.567 (0.501 – 0.633) | 0.564 (0.501 – 0.628) |
| *XGBoost, AUC (95% CI)* | 0.571 (0.506 - 0.635) | 0.547 (0.482 - 0.612) | 0.564 (0.499 - 0.628) | 0.513 (0.450 - 0.576) |

**Table S11: Demographic and clinical features at baseline, for patients correctly classified with high probability.** *Classifications were based on cut-offs suggested as optimal from maximizing Youden’s index, with high probability defined as those in the top/bottom decile of predicted probabilities. P-values were obtained by t-tests for numerical variables and χ^2^-tests for categorical variables.*

|  | **Persistence at one year** | | | **Persistence at three years** | | |
| --- | --- | --- | --- | --- | --- | --- |
|  | *Non-persistent* | *Persistent* | *P* | *Non-persistent* | *Persistent* | *P* |
| *N* | 143 | 197 |  | 175 | 148 |  |
| **Demographics** |  |  |  |  |  |  |
| *Female (%)* | 119 (83%) | 96 (49%) | 1.59 ∙ 10^-10^ | 145 (83%) | 76 (51%) | 2.69 ∙ 10^-9^ |
| *Age (IQR)* | 44 (22) | 70 (10) | 1.58 ∙ 10^-54^ | 32 (15) | 75 (9) | 5.83 ∙ 10^-150^ |
| *Born in Sweden (%)* | 127 (89%) | 177 (90%) | 0.8981 | 165 (94%) | 135 (91%) | 0.3944 |
| *First-degree relative with RA (%)* | 5 (3%) | 6 (3%) | 1 | 5 (3%) | 3 (2%) | 0.9053 |
| **Educational level** |  |  |  |  |  |  |
| *< 9 years (%)* | REF | REF | REF | REF | REF | REF |
| *9 – 12 years (%)* | 69 (48%) | 75 (38%) | 0.0777 | 77 (44%) | 62 (42%) | 0.7883 |
| *≥ 12 years (%)* | 49 (34%) | 55 (28%) | 0.2565 | 76 (43%) | 34 (23%) | 0.0002 |
| **Smoking status** |  |  |  |  |  |  |
| *Never (%)* | REF | REF | REF | REF | REF | REF |
| *Previous (%)* | 14 (10%) | 36 (18%) | 0.0428 | 16 (9%) | 34 (23%) | 0.0011 |
| *Current (%)* | 7 (5%) | 13 (7%) | 0.6703 | 4 (2%) | 7 (5%) | 0.3688 |
| **Healthcare utilization, year before MTX start** |  |  |  |  |  |  |
| *Days hospitalized (IQR; min-max)* | 0 (0; 0-56) | 0 (0; 0-27) | 0.4823 | 0 (0; 0-56) | 0 (0; 0-27) | 0.2390 |
| *Days on disability pension (IQR; min-max)* | 0 (0; 0-365) | 0 (0; 0-365) | 0.0030 | 0 (0; 0-365) | 0 (0; 0-121.5) | 0.0462 |
| *Days on sick leave (IQR; min-max)* | 0 (54; 0-365) | 0 (0; 0-63) | 3.03 ∙ 10^-9^ | 0 (11; 0-365) | 0 (0; 0-107) | 1.06 ∙ 10^-6^ |
| *Prescription expenditures, in SEK (IQR)* | 2503 (3820) | 2038 (2854) | 0.0935 | 1375 (2060) | 2475 (3225) | 0.0557 |
| **Disease characteristics, at MTX start** |  |  |  |  |  |  |
| *Duration, in months (IQR)* | 5 (4) | 4 (4) | 0.1679 | 5 (5) | 4 (4) | 0.0045 |
| *Seropositive (%)* | 101 (72%) | 128 (71%) | 0.3268 | 134 (78%) | 96 (69%) | 0.0284 |
| **Disease activity components at MTX start** |  |  |  |  |  |  |
| *Swollen joint count, 28 joints, (IQR)* | 9 (7) | 6 (6) | 1.33 ∙ 10^-7^ | 9 (8) | 7 (6) | 0.0003 |
| *Tender joint count, 28 joints (IQR)* | 9 (9.5) | 3 (5) | 1.57 ∙ 10^-15^ | 9 (9) | 4 (7) | 1.93 ∙ 10^-10^ |
| *Erythrocyte sedimentation rate (IQR)* | 32 (37) | 22 (24) | 1.36 ∙ 10^-6^ | 20.5 (24) | 26 (30) | 0.1571 |
| *C-reactive protein levels (IQR)* | 28 (62) | 6 (11) | 1.25 ∙ 10^-12^ | 12 (22) | 9 (16) | 0.0249 |
| *Patient Global Health (IQR)* | 76 (21) | 16 (25) | 2.30 ∙ 10^-72^ | 70 (25) | 19 (29) | 9.74 ∙ 10^-57^ |
| **Additional clinical measurements** |  |  |  |  |  |  |
| *Health Assessment Questionnaire (IQR)* | 1.5 (0.75) | 0.38 (0.62) | 1.51 ∙ 10^-45^ | 1.13 (0.75) | 0.75 (0.88) | 2.43 ∙ 10^-9^ |
| *Pain (IQR)* | 76 (21) | 21 (28) | 7.13 ∙ 10^-61^ | 70 (29) | 31 (31) | 6.58 ∙ 10^-29^ |
| **Additional therapies at MTX start** |  |  |  |  |  |  |
| *Corticosteroids (%)* | 97 (75%) | 128 (67%) | 0.6645 | 119 (76%) | 106 (76%) | 0.5592 |
| *Non-steroidal anti-inflammatory drugs (%)* | 52 (40%) | 19 (10%) | 4.96 ∙ 10^-9^ | 55 (35%) | 13 (9%) | 1.31 ∙ 10^-6^ |

**Table S12: Demographics and clinical features at baseline, for those correctly classified with high probability.** *Classifications were based on cut-offs suggested as optimal in maximizing Youden’s index, with high probability defined as those in the top/bottom three deciles of predicted probabilities. P-values were obtained by t-tests for numerical variables and χ^2^-tests for categorical variables.*

|  | **Persistence at one year** | | | **Persistence at three years** | | |
| --- | --- | --- | --- | --- | --- | --- |
|  | *Non-persistent* | *Persistent* | *P* | *Non-persistent* | *Persistent* | *P* |
| *N* | 350 | 583 |  | 474 | 426 |  |
| **Demographics** |  |  |  |  |  |  |
| *Female (%)* | 288 (82%) | 322 (55%) | 7.51 ∙ 10^-17^ | 364 (77%) | 242 (57%) | 2.75 ∙ 10^-10^ |
| *Age (IQR)* | 50 (24) | 67 (13) | 5.34 ∙ 10^-67^ | 42 (19) | 70 (9) | 1.89 ∙ 10^-220^ |
| *Born in Sweden (%)* | 306 (87%) | 531 (91%) | 0.0956 | 417 (88%) | 392 (92%) | 0.0576 |
| *First-degree relative with RA (%)* | 12 (3%) | 20 (3%) | 1.00 | 15 (3%) | 12 (3%) | 0.9127 |
| **Educational level** |  |  |  |  |  |  |
| *< 9 years (%)* | REF | REF | REF | REF | REF | REF |
| *9 – 12 years (%)* | 168 (48%) | 247 (42%) | 0.1078 | 225 (47%) | 178 (42%) | 0.0999 |
| *≥ 12 years (%)* | 104 (30%) | 175 (3%) | 0.9809 | 183 (39%) | 123 (29%) | 0.0026 |
| **Smoking status** |  |  |  |  |  |  |
| *Never (%)* | REF | REF | REF | REF | REF | REF |
| *Previous (%)* | 45 (13%) | 88 (15%) | 0.3955 | 51 (11%) | 86 (20%) | 0.0001 |
| *Current (%)* | 22 (6%) | 31 (5%) | 0.6365 | 21 (4%) | 28 (7%) | 0.2051 |
| **Healthcare utilization, year before MTX start** |  |  |  |  |  |  |
| *Days hospitalized (IQR; min-max)* | 0 (0; 0-56) | 0 (0; 0-27) | 0.0431 | 0 (0; 0-56) | 0 (0) | 0.7809 |
| *Days on disability pension (IQR; min-max)* | 0 (0; 0-365) | 0 (0; 0-365) | 0.0002 | 0 (0; 0-365) | 0 (0) | 0.0003 |
| *Days on sick leave (IQR; min-max)* | 0 (33; 0-365) | 0 (0; 0-113) | 5.43 ∙ 10^-13^ | 0 (22; 0-365) | 0 (0) | 1.09 ∙ 10^-15^ |
| *Prescription expenditures, in SEK (IQR)* | 2032 (3962) | 1913 (2896) | 0.0276 | 1565 (2600) | 2365 (3409) | 0.1601 |
| **Disease characteristics, at MTX start** |  |  |  |  |  |  |
| *Duration, in months (IQR)* | 5 (4) | 5 (4) | 0.0592 | 5 (5) | 4 (4) | 0.0643 |
| *Seropositive (%)* | 256 (76%) | 402 (74%) | 0.1989 | 376 (81%) | 263 (67%) | 9.9162 ∙ 10^-9^ |
| **Disease activity components at MTX start** |  |  |  |  |  |  |
| *Swollen joint count, 28 joints, (IQR)* | 8 (8) | 6 (7) | 1.52 ∙ 10^-6^ | 7 (8) | 7 (7) | 0.3629 |
| *Tender joint count, 28 joints (IQR)* | 9 (9) | 4 (6) | 1.03 ∙ 10^-21^ | 8 (8) | 6 (7) | 8.4285 ∙ 10^-8^ |
| *Erythrocyte sedimentation rate (IQR)* | 32 (38) | 22 (25) | 5.97 ∙ 10^-11^ | 24 (28) | 27 (28) | 0.1571 |
| *C-reactive protein levels (IQR)* | 21 (44) | 7 (14) | 2.03 ∙ 10^-19^ | 11 (28) | 10 (19) | 0.0019 |
| *Patient Global Health (IQR)* | 71 (25) | 30 (31) | 1.81 ∙ 10^-113^ | 69 (31) | 31 (34) | 2.18 ∙ 10^-76^ |
| **Additional clinical measurements** |  |  |  |  |  |  |
| *Health Assessment Questionnaire (IQR)* | 1.38 (0.62) | 0.63 (0.75) | 2.93 ∙ 10^-68^ | 1.13 (0.75) | 0.88 (0.87) | 4.33 ∙ 10^-15^ |
| *Pain (IQR)* | 72 (26) | 34 (30) | 1.90 ∙ 10^-86^ | 69 (34) | 40 (37) | 4.18 ∙ 10^-41^ |
| **Additional therapies at MTX start** |  |  |  |  |  |  |
| *Corticosteroids (%)* | 239 (77%) | 359 (66%) | 0.0458 | 303 (71%) | 272 (71%) | 1.00 |
| *Non-steroidal anti-inflammatory drugs (%)* | 103 (33%) | 94 (17%) | 2.15 ∙ 10^-6^ | 137 (32%) | 50 (13%) | 3.97 ∙ 10^-10^ |

|  | **Persistence at one year** | | | **Persistence at three years** | | |
| --- | --- | --- | --- | --- | --- | --- |
|  | *Persistent* | *Non-persistent* | *P* | *Persistent* | *Non-persistent* | *P* |
| *N* | 583 | 350 |  | 426 | 474 |  |
| **Demographics** |  |  |  |  |  |  |
| *Female (%)* | 322 (55%) | 288 (82%) | 7.51 ∙ 10^-17^ | 242 (57%) | 364 (77%) | 2.75 ∙ 10^-10^ |
| *Age (IQR)* | 67 (13) | 50 (24) | 5.34 ∙ 10^-67^ | 70 (9) | 42 (19) | 1.89 ∙ 10^-220^ |
| *Born in Sweden (%)* | 531 (91%) | 306 (87%) | 0.0956 | 392 (92%) | 417 (88%) | 0.0576 |
| *First-degree relative with RA (%)* | 20 (3%) | 12 (3%) | 1.00 | 12 (3%) | 15 (3%) | 0.9127 |
| **Educational level** |  |  |  |  |  |  |
| *< 9 years (%)* | REF | REF | REF | REF | REF | REF |
| *9 – 12 years (%)* | 247 (42%) | 168 (48%) | 0.1078 | 178 (42%) | 225 (47%) | 0.0999 |
| *≥ 12 years (%)* | 175 (3%) | 104 (30%) | 0.9809 | 123 (29%) | 183 (39%) | 0.0026 |
| **Smoking status** |  |  |  |  |  |  |
| *Never (%)* | REF | REF | REF | REF | REF | REF |
| *Previous (%)* | 88 (15%) | 45 (13%) | 0.3955 | 86 (20%) | 51 (11%) | 0.0001 |
| *Current (%)* | 31 (5%) | 22 (6%) | 0.6365 | 28 (7%) | 21 (4%) | 0.2051 |
| **Healthcare utilization, year before MTX start** |  |  |  |  |  |  |
| *Days hospitalized (IQR; min-max)* | 0 (0; 0-27) | 0 (0; 0-56) | 0.0431 | 0 (0) | 0 (0; 0-56) | 0.7809 |
| *Days on disability pension (IQR; min-max)* | 0 (0; 0-365) | 0 (0; 0-365) | 0.0002 | 0 (0) | 0 (0; 0-365) | 0.0003 |
| *Days on sick leave (IQR; min-max)* | 0 (0; 0-113) | 0 (33; 0-365) | 5.43 ∙ 10^-13^ | 0 (0) | 0 (22; 0-365) | 1.09 ∙ 10^-15^ |
| *Prescription expenditures, in SEK (IQR)* | 1913 (2896) | 2032 (3962) | 0.0276 | 2365 (3409) | 1565 (2600) | 0.1601 |
| **Disease characteristics, at MTX start** |  |  |  |  |  |  |
| *Duration, in months (IQR)* | 5 (4) | 5 (4) | 0.0592 | 4 (4) | 5 (5) | 0.0643 |
| *Seropositive (%)* | 402 (74%) | 256 (76%) | 0.1989 | 263 (67%) | 376 (81%) | 9.9162 ∙ 10^-9^ |
| **Disease activity components at MTX start** |  |  |  |  |  |  |
| *Swollen joint count, 28 joints, (IQR)* | 6 (7) | 8 (8) | 1.52 ∙ 10^-6^ | 7 (7) | 7 (8) | 0.3629 |
| *Tender joint count, 28 joints (IQR)* | 4 (6) | 9 (9) | 1.03 ∙ 10^-21^ | 6 (7) | 8 (8) | 8.4285 ∙ 10^-8^ |
| *Erythrocyte sedimentation rate (IQR)* | 22 (25) | 32 (38) | 5.97 ∙ 10^-11^ | 27 (28) | 24 (28) | 0.1571 |
| *C-reactive protein levels (IQR)* | 7 (14) | 21 (44) | 2.03 ∙ 10^-19^ | 10 (19) | 11 (28) | 0.0019 |
| *Patient Global Health (IQR)* | 30 (31) | 71 (25) | 1.81 ∙ 10^-113^ | 31 (34) | 69 (31) | 2.18 ∙ 10^-76^ |
| **Additional clinical measurements** |  |  |  |  |  |  |
| *Health Assessment Questionnaire (IQR)* | 0.63 (0.75) | 1.38 (0.62) | 2.93 ∙ 10^-68^ | 0.88 (0.87) | 1.13 (0.75) | 4.33 ∙ 10^-15^ |
| *Pain (IQR)* | 34 (30) | 72 (26) | 1.90 ∙ 10^-86^ | 40 (37) | 69 (34) | 4.18 ∙ 10^-41^ |
| **Additional therapies at MTX start** |  |  |  |  |  |  |
| *Corticosteroids (%)* | 359 (66%) | 239 (77%) | 0.0458 | 272 (71%) | 303 (71%) | 1.00 |
| *Non-steroidal anti-inflammatory drugs (%)* | 94 (17%) | 103 (33%) | 2.15 ∙ 10^-6^ | 50 (13%) | 137 (32%) | 3.97 ∙ 10^-10^ |

**Table S13: Refitted prediction model quality.** *Model quality measures in predicting persistence at one and three years, respectively, based on elastic net regression trained with non-genetic characteristics and PRS; AUC confidence intervals are computed through 2000 stratified bootstrap estimates.*

|  | *Persistence at one year* | *Persistence at three years* |
| --- | --- | --- |
| *AUC (95% CI)* | 0.666 (0.643-0.689) | 0.644 (0.622-0.666) |
| **Cut-off specific measures** |  |  |
| *Youden J-statistic (cut-off)* | 0.23 (0.65) | 0.21 (0.45) |
| *Sensitivity* | 0.76 | 0.70 |
| *Specificity* | 0.46 | 0.50 |
| *Positive predictive value* | 0.75 | 0.55 |
| *Negative predictive value* | 0.49 | 0.66 |
| **Measures of calibration quality** |  |  |
| *Weak calibration error* | < 1e-6 | < 1e-6 |
| *Calibration intercept error* | 0.001 | 0.046 |
| *Calibration slope error* | -0.001 | -0.002 |

**SUPPLEMENTARY FIGURES**

**Figure S1: Supervised learning pipeline**. *Flowchart visualization of the nested cross-validation loop as implemented in the underlying supervised learning pipeline. The green box visualizes the outer loop, whereas the blue box visualizes the inner loop, repeated within each of the five segments observable within the green box.*

TEST

TRAIN

TEST

TRAIN

TRAIN

TRAIN

TEST

TRAIN

TRAIN

TEST

TRAIN

TRAIN

TEST

TRAINING DATA

TRAIN

TRAIN

TRAIN

…

**FIGURE S2: Graphical visualization of model calibration for persistence at one year**. *Flexible loess-smoothed curve of fitted probabilities on observed labels, with each point and error bar representing deciles of fitted probabilities. Diagonal line represents expected distribution. Both models are trained with elastic net regression.*

**FIGURE S3: Graphical visualization of model calibration for persistence at three years**. *Flexible loess-smoothed curve of fitted probabilities on observed labels, with each point and error bar representing deciles of fitted probabilities. Diagonal line represents expected distribution. Both models are trained with elastic net regression.*

**REFERENCES**

1.	Westerlind, H., et al., *Remission, response, retention and persistence to treatment with disease-modifying agents in patients with rheumatoid arthritis: a study of harmonised Swedish, Danish and Norwegian cohorts.* RMD Open, 2023. **9**(3).

2.	Stolt, P., et al., *Quantification of the influence of cigarette smoking on rheumatoid arthritis: results from a population based case-control study, using incident cases.* Ann Rheum Dis, 2003. **62**(9): p. 835-41.

3.	Eriksson, J.K., J. Askling, and E.V. Arkema, *The Swedish Rheumatology Quality Register: optimisation of rheumatic disease assessments using register-enriched data.* Clin Exp Rheumatol, 2014. **32**(5 Suppl 85): p. S-147-9.

4.	SRQ. *Visualization and analysis platform (VAP)*. 2024 [cited 2024 19/6].

5.	Ludvigsson, J.F., et al., *The Swedish personal identity number: possibilities and pitfalls in healthcare and medical research.* Eur J Epidemiol, 2009. **24**(11): p. 659-67.

6.	Ludvigsson, J.F., et al., *Registers of the Swedish total population and their use in medical research.* Eur J Epidemiol, 2016. **31**(2): p. 125-36.

7.	Ludvigsson, J.F., et al., *The longitudinal integrated database for health insurance and labour market studies (LISA) and its use in medical research.* Eur J Epidemiol, 2019. **34**(4): p. 423-437.

8.	Ekbom, A., *The Swedish Multi-generation Register.* Methods Mol Biol, 2011. **675**: p. 215-20.

9.	Ludvigsson, J.F., et al., *External review and validation of the Swedish national inpatient register.* BMC Public Health, 2011. **11**: p. 450.

10.	Wettermark, B., et al., *The new Swedish Prescribed Drug Register--opportunities for pharmacoepidemiological research and experience from the first six months.* Pharmacoepidemiol Drug Saf, 2007. **16**(7): p. 726-35.

11.	Genomes Project, C., et al., *A global reference for human genetic variation.* Nature, 2015. **526**(7571): p. 68-74.

12.	Delaneau, O., J.F. Zagury, and J. Marchini, *Improved whole-chromosome phasing for disease and population genetic studies.* Nat Methods, 2013. **10**(1): p. 5-6.

13.	Howie, B., et al., *Fast and accurate genotype imputation in genome-wide association studies through pre-phasing.* Nat Genet, 2012. **44**(8): p. 955-9.

14.	Stekhoven, D.J. and P. Bühlmann, *MissForest--non-parametric missing value imputation for mixed-type data.* Bioinformatics, 2012. **28**(1): p. 112-8.

15.	Coombes, B.J., et al., *A principal component approach to improve association testing with polygenic risk scores.* Genet Epidemiol, 2020. **44**(7): p. 676-686.

16.	Prive, F., et al., *Making the Most of Clumping and Thresholding for Polygenic Scores.* Am J Hum Genet, 2019. **105**(6): p. 1213-1221.

17.	Van Calster, B., et al., *Calibration: the Achilles heel of predictive analytics.* BMC Med, 2019. **17**(1): p. 230.

18.	Lim, L.J., et al., *Machine learning using genetic and clinical data identifies a signature that robustly predicts methotrexate response in rheumatoid arthritis.* Rheumatology (Oxford), 2022. **61**(10): p. 4175-4186.

19.	Myasoedova, E., et al., *Toward Individualized Prediction of Response to Methotrexate in Early Rheumatoid Arthritis: A Pharmacogenomics-Driven Machine Learning Approach.* Arthritis Care Res (Hoboken), 2-022. **74**(6): p. 879-888.
